# Early responses of primary human and bovine monocytes, monocytic THP-1 cells and THP-1 cell-derived macrophages to vital *Toxoplasma gondii* tachyzoites

**DOI:** 10.3389/fimmu.2025.1683634

**Published:** 2025-10-30

**Authors:** Dominik Hanke, Zahady D. Velásquez, Kathrin Büttner, Andreas Krueger, Ralf Ross, Andreas Hecker, Sybille Mazurek, Veronika Grau, Anja Taubert, Carlos Hermosilla, Katrin Richter, Iván Conejeros

**Affiliations:** ^1^ Institute of Veterinary Physiology and Biochemistry, Justus Liebig University Giessen, Giessen, Germany; ^2^ Institute of Parasitology, Biomedical Research Center Seltersberg, Justus Liebig University Giessen, Giessen, Germany; ^3^ Unit for Biomathematics and Data Processing, Justus Liebig University Giessen, Giessen, Germany; ^4^ Institute of Molecular Immunology, Biomedical Research Center Seltersberg, Justus Liebig University Giessen, Giessen, Germany; ^5^ Laboratory of Experimental Surgery, Department of General and Thoracic Surgery, German Center for Lung Research (DZL), Cardiopulmonary Institute (CPI), Justus Liebig University Giessen, Giessen, Germany; ^6^ Department of Natural Sciences, Bonn-Rhein-Sieg University of Applied Sciences, Rheinbach, Germany

**Keywords:** *Toxoplasma gondii*, mononuclear phagocytes, extracellular traps, cytokines, early innate immunity

## Abstract

**Introduction:**

Different innate immune cell types are known to release extracellular traps (ETs) in response to invasive pathogens, including parasites. These ETs function to trap, immobilize, and eventually kill pathogens. In line with this, monocytes and macrophages have been shown to release ETs, known as monocyte/macrophage extracellular traps (METs). *Toxoplasma gondii* (*T. gondii*) is an apicomplexan zoonotic parasite that infects humans and homeothermic animals. While most studies have focused on prolonged exposure of immune cells to *T. gondii*, this study characterized the early innate immune reaction of mononuclear phagocytes to vital *T. gondii* tachyzoites.

**Methods:**

Primary human and bovine monocytes, monocytic THP-1 cells, and THP-1 cell-derived macrophages (M0-, M1-, and M2-like) were exposed to *T. gondii* tachyzoites for 4 h. Scanning electron microscopy (SEM), transmission electron microscopy (TEM), immunofluorescencemicroscopy, and confocal microscopy were used to visualize cell activation and the presence of METs. Additionally, the release of pro-inflammatory cytokines interleukin (IL)-1β and IL-6, and expression of Toll-like receptor (TLR) 2 and TLR4 were analyzed.

**Results and discussion:**

Microscopic analysis illustrated the activation of all cell types tested within 4 h of exposure to *T. gondii* tachyzoites. Numerous tachyzoites were found intracellularly in THP-1 cell-derived M1-like macrophages. Furthermore, the co-localization of extracellular DNA (extDNA) and histones in extracellular web-like fibers proved classical characteristics of extruded *T. gondii*-induced METs, although this was a rare event. In primary human monocytes, an increased release of IL-1β and IL-6 was observed following exposure to *T. gondii* tachyzoites. When co-stimulated with lipopolysaccharide (LPS), primary human monocytes showed an enhanced release of IL-1β and IL-6 in response to *T. gondii*. In contrast to monocytic THP-1 cells, THP-1 cell-derived M1-like macrophages released IL-1β in response to *T. gondii* tachyzoite exposure. When additionally stimulated by LPS, all THP-1 cell-derived macrophages showed an enhanced release of IL-1β, and monocytic THP-1 cells an increased release of IL-6 in response to *T. gondii* tachyzoites. This study provides insights into the early innate immune response of human and bovine mononuclear phagocytes to *T. gondii*. While cytokine secretion was prominent, MET formation was rare in the early response (i.e. < 4 h of exposure) to *T. gondii* tachyzoites.

## Introduction

*Toxoplasma gondii* (*T. gondii*) is a polyxenous zoonotic apicomplexan parasite able to infect warm-blooded mammals and birds worldwide (1). *T. gondii* is the etiological agent of human toxoplasmosis and it is estimated that one-third of the global population is seropositive for *T. gondii* (2). In humans, toxoplasmosis is particularly problematic in children after congenital transmission (i.e. prenatal toxoplasmosis) and in opportunistic infections of immunocompromised patients (i.e. postnatal toxoplasmosis) (3, 4). *T. gondii* is the only representative species of its genus and belongs to the class Sporozoa (subclass Coccidia, family Sarcocystidae). The life cycle of *T. gondii* comprises distinct parasitic stages. Infective parasitic stages are sporozoites within sporulated oocysts found in felid feces. Moreover, male and female gametocytes (exclusively form in the intestine of felines, acting as definitive hosts), fast replicating tachyzoites and bradyzoites in tissue cysts of intermediate hosts are found. During the acute phase of toxoplasmosis, fast replicating tachyzoites proliferate within all nucleated cell types, whereas bradyzoites, i.e. the slow replicating stages, develop within cysts and are primarily found in muscles, spleen, liver and brain. These stages propagate the chronic persistence of *T. gondii*. The main transmission routes of *T. gondii* to humans are: *i)* ingestion of undercooked meat containing tissue cysts, *ii)* consumption of food- and/or water contaminated with sporulated oocysts, *iii)* congenital transmission, *iv)* administration of blood transfusions containing tachyzoites, and *v)* organ transplants containing either tissue cysts or tachyzoites (2, 5).

Within the mammalian host innate immune system, mononuclear phagocytes like monocytes and macrophages play a crucial role in the initiation of adaptive immunity by acting as first responders to infection in the vasculature and tissue (6, 7). After polymorphonuclear neutrophils (PMN), monocytes are the second most abundant phagocytes in the blood, comprising 10% of the peripheral white blood cells in humans and 4% in cattle (8, 9). Besides macrophages and dendritic cells (DC), monocytes form part of the mononuclear phagocyte system (MPS) and are characterized by their rapid recruitment in response to pathogen infection or tissue injury (9, 10). Classical monocyte- or macrophage-derived effector mechanisms, include phagocytosis, chemokine- [e.g. chemoattractant protein-1 (CCL2)] and cytokine secretion [e.g. interleukin (IL)-1β and IL-6)], reactive oxygen species (ROS) production, and expression of a wide range of Toll-like receptors (TLRs) and purinergic receptors (e.g. P2X7) (11–16). Additionally, mammalian monocytes and macrophages are capable to release extracellular traps (METs) (17–24), an early defense mechanism which was first observed in PMN and named neutrophil extracellular traps (NETs) (25). As shown for extruded NETs, METs are mainly composed of extracellular DNA (extDNA) and proteins like citrullinated histones (H1, H2A/H2B, H3, H4) and myeloperoxidase (MPO). METs function to trap, immobilize, and eventually kill pathogens, including parasites such as *Besnoitia besnoiti* (26), *Cryptosporidium parvum* (23), *Eimeria ninakohlyakimovae* (19) and *Neospora caninum* (21). In addition, the release of METs has been described in response to human sperm *in vitro* and *ex vivo* in male patients with leukocytospermia (27). Nonetheless, only one study so far reported on *T. gondii*-induced MET release, namely in monocytes of harbour seals (*Phoca vitulina*) (18), thereby proving that this defense mechanism seems to be highly conserved, as also assumed for NET formation (28). MET release is also induced by chemical activators like A23187 (calcium ionophore), phorbol 12-myristate 13-acetate (PMA), platelet activating factor (PAF) (20, 24), zymosan (18–21, 26), or gold nanoparticles (29).

Monocytic THP-1 cells and THP-1 cell-derived-macrophages are a widely accepted model for human mononuclear phagocytes to study immunological mechanisms *in vitro* (30). THP-1 cells were originally isolated from a patient with acute myeloid leukemia (30–32). Monocytic THP-1 cells can be differentiated into non-polarized M0-like macrophages, pro-inflammatory M1-like macrophages and anti-inflammatory M2-like macrophages, providing the opportunity to directly compare all four cell types *in vitro* (16, 30, 33).

Monocytic THP-1 cells and THP-1 cell-derived macrophages have been shown to release METs in response to a variety of chemical activators and stimuli [e.g. tunicamycin (34), PMA (35), A23187, HOCl, nigericin, tumor necrosis factor (TNF)-α (36), zymosan (37), murine extracellular cold-inducible RNA-binding protein (eCIRP) (38), and aflatoxin B1 (39)] as well as parasite [e.g. *Trichomonas vaginalis* (37)] and bacteria [e.g. *Escherichia coli* (17), *Streptococcus agalactiae* (40), and *Mycobacterium massiliense* (41)].

An important function of mononuclear phagocytes is the release of cytokines like IL-1β and IL-6 in response to diverse danger- or pathogen-associated molecular patterns (DAMPs, PAMPs) (42–45). IL-1β and IL-6 have distinct roles in the inflammatory process. IL-1β is a primary initiator of innate immune reaction and key player in host defense against infections, while the pro- and anti-inflammatory IL-6 amplifies and modulates inflammatory responses (44). IL-1β is processed from a precursor by caspase cleavage to become biologically active and is then secreted. The mere induction of pro-IL-1β expression (priming step) is inefficient to induce secretion (44, 46). Consecutive second PAMPs or DAMPs are required to induce the NOD- LRR- and pyrin domain-containing protein 3 (NLRP3) inflammasome-dependent processing, and secretion of mature IL-1β (45). IL-6 is secreted by mononuclear phagocytes in response to PAMPs (44, 46). These PAMPs bind to pattern recognition receptors (PRRs) such as TLR2.

In mammals, 13 TLRs are known. These are located on the plasma membrane and in intracellular compartments, where they activate signaling cascades to produce molecules and cytokines to fight against pathogens including *T. gondii* (47, 48). TLR2 and TLR4 have been shown to be involved in immune responses against *T. gondii*, with TLR2 recognizing the parasitic surface profilin or glycosylphosphatidylinositol (GPI) proteins, and stimulating the production of IL-12, while TLR4 plays a supportive role, especially at high infectious doses, as shown for several mouse models (47).

The role of IL-1β and IL-6 in *T. gondii*-induced immune responses is controversial, possibly due to the use of different parasite strains (e.g. type I [RH], type II [Prugniaud, ME49], type III [CEP], or atypical strains such as TgChBrUD2), reflecting the considerable genetic variability of *T. gondii*. This strain diversity is reflected in pronounced differences in virulence and immune modulation among strains (49, 50), which may explain the inconsistent findings. A key factor contributing to this variability is partly driven by strain-specific polymorphic effector proteins that differentially modulate host immune signaling pathways, thereby influencing cytokine responses (51, 52). In line with this, Gov et al., 2013 and Guimaraes Gois et al., 2022 demonstrated strain-specific cytokine profiles (53, 54). While some studies on primary human monocytes showed a *T. gondii* tachyzoite-induced increased release of IL-1β after 4, 16 and 24 h (53, 55–58), Liam et al., 2018 found no impact of tachyzoite exposure on lipopolysaccharide (LPS)-induced IL-1β release (56). The same applies for IL-6. Pelloux et al., 1994 and Tombácz et al., 2018 found no impact of *T. gondii* tachyzoites or profilin on the release of IL-6 by primary monocytes after 24 h exposure (59, 60), whereas Salazar Gonzalez et al., 2014 have found an impact of *T. gondii* profilin (61). Further studies provide evidence for a parasite-induced release of macrophage migration inhibitory factor (MIF), IL-1β, IL-6 and IL-12 by monocytic THP-1 cells (53, 55, 62, 63), and a fostered IL-1β and MIF secretion by M0-like macrophages (54, 64).

Of note, the above described studies either focused on the release of METs (18), production of IL-1β and IL-6 (53–61, 63, 64) or the expression of TLR2 and TLR4 (47, 48).

Here we studied in a comprehensive systematic manner the effect of *T. gondii* tachyzoites on the acute and early phase response of primary human and bovine monocytes as well as monocytic THP-1 cells and THP-1 cell-derived macrophages, including the release of METs, the production of pro-inflammatory cytokines IL-1β and IL-6, and the expression of the surface receptors TLR2 and TLR4.

## Materials and methods

### THP-1 cell culture

Monocytic THP-1 cells (Leibniz Institute, DSMZ-German Collection of Microorganisms and Cell Cultures GmbH, Braunschweig, Germany, Cat#ACC16) were cultured in T-75 cell culture flasks (Sarstedt, Nümbrecht, Germany, Cat#83.3911.002) in Roswell Park Memorial Institute (RPMI) 1640 medium (Sigma-Aldrich, Merck, Darmstadt, Germany, Cat#R8758) supplemented with 10% fetal calf serum (FCS) Xtra (Capricorn Scientific, Ebsdorfergrund, Germany, Cat#FBS-16A), 1% penicillin-streptomycin [50 U/ml penicillin, 50 µg/ml streptomycin] (Gibco™, Thermo Fisher Scientific, Waltham, Massachusetts, USA, Cat#151401222), and transferred to 12-well plates (Greiner Bio-One, Kremsmünster, Austria, Cat#655180) or 96-well plates (Greiner Bio-One, Cat#655180) on the day of the experiment. To generate THP-1 cell-derived macrophages of M0-, M1-, and M2-like phenotypes, cells were seeded in T-75 cell culture flasks (6 x 10^6^ cells/flask) with RPMI-1640 medium. Cells were differentiated using 50 nM PMA (Thermo Fisher Scientific, Cat#P1585) according to a previously described protocol (16) resulting in cells with macrophage-like properties, such as increased cell adherence and a reduced proliferation rate compared to monocytic THP-1 cells (65). The protocol for the polarization to M1-like macrophages has also been published (16). Briefly, cells were cultured in medium supplemented with 10 ng/ml LPS (*Escherichia coli*, O111:B4, Sigma-Aldrich, Merck, Cat#L2630) and 10 ng/ml interferon (IFN)-γ (R&D Systems, Minneapolis, USA, Cat#285-IF) for 48 h (16). For polarization to M2-like macrophages, the medium was supplemented with 20 ng/ml IL-4 (Preprotec, Thermo Fisher Scientific, Cat#200-04) and 20 ng/ml IL-13 (Gibco™, Preprotec, Thermo Fisher Scientific, Cat#200-13). On day 5 of differentiation, cells in T-75 cell culture flasks were washed with Dulbecco’s phosphate buffered saline (PBS, Sigma-Aldrich, Merck, Cat#D8537), detached using TrypLE™ Express (Gibco™, Thermo Fisher Scientific, Cat#12605010) according to the manufacturer’s protocol, transferred to Eppendorf tubes (Sarstedt, Cat#72.691), 8-well cell culture chambers (Sarstedt, Cat#94.6170.802), 12-well plates or 96-well plates and cultured in Dulbecco’s modified Eagle’s medium (DMEM) without glucose, glutamine and phenol red (Gibco™, Thermo Fisher Scientific, Cat#A1443001) supplemented with 10% heat-inactivated FCS Xtra, 2 mM D-glucose (Carl Roth, Karlsruhe, Germany, Cat#X997.1), 1 mM L-glutamine (Sigma-Aldrich, Merck, Cat#G3126), 1% penicillin-streptomycin. For all experiments the same batch of FCS Xtra was used and heat-inactivation was done according to the manufacturer’s protocol, as FCS contains active deoxyribonuclease (DNases) that can degrade extracellular traps (66).

### Isolation of primary human and bovine monocytes

Primary human monocytes were obtained by positive and negative selection protocols. For positive selection, buffy coats from healthy female and male donors (n = 6) of the Center of Transfusion Medicine and Hemotherapy (JLU Giessen) and the University Hospital of Giessen and Marburg (UKGM), were used. The protocol for positive selection of primary human monocytes was based on a previously published method (67). Briefly, 15 ml of buffy coats were diluted 1:1 with RPMI-1640 medium (Sigma-Aldrich, Cat#R5886), supplemented with 5% FCS (Biochrom GmbH, Berlin, Germany, Cat#S0115), 2 mM L-glutamine (Sigma-Aldrich, Merck, Darmstadt, Germany, Cat#G7513), 1 mM sodium pyruvate (Sigma-Aldrich, Merck, Cat#S8636), 1 mM minimum essential medium (MEM) non-essential amino acid solution (Sigma-Aldrich, Merck, Cat#M7145) and 1% penicillin-streptomycin (Sigma-Aldrich, Merck, Cat#P4333), transferred to 15 ml Biocoll^®^ separation solution (Biochrom GmbH, Cat#L6115, density: 1.077 g/ml), and centrifuged [2500 x *g*, without brake, 30 min, room temperature (RT)]. The ring containing peripheral blood mononuclear cells (PBMCs) was carefully collected and cells were washed three times by filling up to a volume of 30 ml with MACS^®^ separation buffer (Miltenyi Biotec GmbH, Bergisch Gladbach, Germany, Cat#130-091-222) containing 2% bovine serum albumin (BSA; Miltenyi Biotec GmbH, Cat#130-091-376) followed by centrifugation (1500 x *g*, 7 min, 4 °C). The supernatant was discarded and the PBMC pellet was resuspended in 5 ml MACS^®^ separation buffer containing 2% BSA and subjected to positive selection for CD14^+^ monocytes using immunomagnetic human CD14^+^ MicroBeads^®^ (Miltenyi Biotec GmbH, Cat#130-050-201). Briefly, 2.5 x 10^8^ PBMCs and 500 µl CD14^+^ MicroBeads^®^ were incubated in a total volume of 2.5 ml MACS^®^ separation buffer containing 2% BSA for 15 min at 4 °C. CD14^+^ monocytes were isolated using LS^®^ columns (Miltenyi Biotec GmbH, Cat#130-042-401) and QuadroMACS™ separators (Miltenyi Biotec GmbH, Cat#130-090-976) according to the manufacturer’s protocol. Isolated human monocytes were resuspended in cold RPMI-1640 medium, supplemented with 5% FCS, 2 mM L-glutamine, 1 mM sodium pyruvate, 1 mM MEM non-essential amino acid solution and 1% penicillin-streptomycin (all Sigma-Aldrich) and stored on ice until experiments.

For negative selection, human peripheral blood was obtained from healthy female and male adult donors (n = 6) via antecubital venipuncture and collected in a heparinized syringe (Heparin-Natrium-5000-ratiopharm^®^, ratiopharm, Ulm, Germany) containing 1 mM ethylenediaminetetraacetic acid (EDTA; Applichem, Darmstadt, Germany, Cat#A-1105) per one ml blood. Monocytes were isolated from whole blood using the RosetteSep™ human Monocyte Enrichment Cocktail (Stemcell Technologies, Cologne, Germany, Cat#15068) and Lymphoprep™ density gradient (Stemcell Technologies, Cat#18060), following to the manufacturer’s protocol. Monocytes were resuspended in cold RPMI-1640 medium (Capricorn Scientific, Cat#RPMI-ADV), supplemented with 1% penicillin-streptomycin (Gibco™, Thermo Fisher Scientific, Cat#15140-122) and stored on ice until performing experiments. The purity of monocytes was confirmed to be > 70% by flow cytometry analysis (FCA) as previously described (16).

For the isolation of primary bovine monocytes, healthy adult dairy cows (n = 6) served as blood donors. Peripheral blood was obtained by jugular venipuncture and collected in heparinized tubes (Kabe Labortechnik GmbH, Nümbrecht-Elsenroth, Germany, Cat#041551). The modified protocol for the isolation of primary bovine monocytes was based on previously published methods (23, 68, 69). In brief, 50 ml blood was centrifuged (1260 x *g*, without brake, 30 min, RT). Then 10 ml plasma plus the ring containing PBMCs was collected. The plasma phase was filtered through a 0.45 µm filter (Sarstedt, Cat#83.1826) and incubated for 1 h in a 2% gelatin-coated T-75 flask at 37 °C and 5% CO_2_. For gelatin coating, the T-75 flask was incubated the day before with 2% gelatin solution (Sigma-Aldrich, Merck, Cat#G1890) for 2 h at 37 °C and 5% CO_2_, the supernatant was removed, and the T-75 flask was dried overnight in the same conditions. The PBMCs were resuspended in 30 ml PBS containing 0.02% EDTA, transferred to 15 ml Histopaque^®^-1077 separation solution (Sigma-Aldrich, Merck, Cat#10771, density: 1.077 g/ml) and centrifuged (1500 x *g*, without brake, 30 min, 4 °C). The ring containing PBMCs was collected, and cells were washed two times by filling up to a volume of 50 ml with PBS containing 0.02% EDTA followed by centrifugation (400 x *g*, 7 min, 4 °C). The supernatant was discarded and PBMCs were resuspended in 10 ml of pre-warmed RPMI-1640 medium (Sigma-Aldrich, Merck, Cat#R8758) supplemented with 1% penicillin and streptomycin (Gibco™, Thermo Fisher Scientific, Cat#151401222). The supernatant of incubated plasma in the T-75 flask was removed and the T-75 flask was washed once with pre-warmed RPMI-1640 medium supplemented with 1% penicillin-streptomycin. The PBMC suspension was transferred to the washed T-75 flask and incubated for 1 h at 37 °C and 5% CO_2_. Non-adherent cells were removed by rinsing the flask once with pre-warmed RPMI-1640 medium supplemented with 1% penicillin-streptomycin. Monocytes were detached by addition of 10 ml 10 mM EDTA suspended in Hanks’ Balanced Salt Solution (HBSS) without sodium bicarbonate, calcium and magnesium (Corning^®^, USA, New York, Cat#20-021-CVR) for 10 min at 37 °C and 5% CO_2_, centrifuged (400 x *g*, 10 min, 4°C), resuspended in cold RPMI-1640 medium supplemented with 1% penicillin-streptomycin, and stored on ice. A commercially available mouse anti-bovine CD172a antibody (Bio-Rad Laboratories, Feldkirchen, Germany, Cat#MCA2041GA) was used to stain primary bovine monocytes, resulting in a purity of > 90% for CD172^+^ monocytes (**Supplementary Figure S1**).

### *In vitro* culture of *Toxoplasma gondii* tachyzoites

Vital tachyzoites of *T. gondii* (RH strain) were isolated from continuous passages on African green monkey kidney epithelial (MARC)-145 cells using DMEM (Sigma-Aldrich, Merck, Cat#D6429) supplemented with 5% FCS, 1% penicillin-streptomycin (Gibco™, Thermo Fisher Scientific, Cat#151401222). *T. gondii* tachyzoites released from host cell cultures were seperated from cell detritus by centrifugation (460 x *g*, 1 min, RT). Supernatants containing *T. gondii* tachyzoites, were filtered (5 µm pore sized filters, Satorius, Göttingen, Germany, Minisart^®^, Cat#17594-K) and pelleted (460 x *g*, 5 min, RT). The harvested *T. gondii* tachyzoites were counted in a Neubauer chamber, suspended in medium, and used for co-incubation with primary human or bovine monocytes as well as monocytic THP-1 cells or THP-1 cell-derived M0-, M1-, and M2-like macrophages. All experiments were performed at 37 °C and 5% CO_2_ atmosphere, with a multiplicity of infection (MOI) of 1:4 (cells: parasites), and a co-incubation period of 4 h.

### Scanning electron microscopy

In total, 2.5 x 10^5^ primary human or bovine monocytes as well as monocytic THP-1 cells or THP-1 cell-derived M0-, M1-, and M2-like macrophages were either left untreated or co-cultured for 4 h with 1 x 10^6^ *T. gondii* tachyzoites on 10 mm coverslips (Carl Roth, Cat#KHX4.1) pre-coated with 0.01% poly-L-lysine (Sigma-Aldrich, Cat#4707) in 12-well plates. After incubation, cells were fixed in 2.5% glutaraldehyde (Sigma-Aldrich, Merck, Cat#G6257), post-fixed in 1% osmium tetroxide (Polysciences Inc., Warrington, Pennsylvania, USA, Cat#0223B-10), washed in purified water critical point-dried using CO_2_ treatment, and sputtered with gold particles. The samples were analyzed using a Philips XL30 microscope (Amsterdam, Netherlands) at the Institute of Anatomy and Cell Biology (JLU Giessen). Brightness and contrast were processed in a standardized way using Image J software, Fiji Is Just ImageJ (FIJI) version (70). The percentage of protrusion positive cells was calculated for each primary monocyte donor, using a cell range of 282 to 796 for human cells and 465 to 1871 for bovine cells. Cells were classified as protrusion positive by the presence of filopodia and cytonemes.

### Pappenheim staining

1 x 10^5^ THP-1 cell-derived M0-, M1-, and M2-like macrophages were either left untreated or co-cultured with 4 x 10^5^ *T. gondii* tachyzoites in 8-well cell culture chambers for 4 h and fixed with 4% formaldehyde (Carl Roth, Cat#CP10.4) diluted in PBS for 15 min at RT. Samples were gently washed three times with PBS. For staining, the cells were treated with May-Grünwald’s eosine-methylene blue solution (Sigma-Aldrich, Merck, Cat#1.01424) for 2 min, distilled water for 5 min, Giemsa’s azur eosin methylene blue solution (1:20, Sigma-Aldrich, Merck, Cat#1.09204) for 15 min, distilled water for 10 min, and air dried at RT. Specimens were documented using a Leica DM IL LED microscope equipped with a Leica Flexcam C3 (Wetzlar, Germany). Brightness and contrast were processed in a standardized way using Image J software, FIJI version (70).

### Transmission electron microscopy

1 x 10^7^ M1-like macrophages were either left untreated or co-cultured with 4 x 10^7^ *T. gondii* tachyzoites in an Eppendorf tube for 4 h and centrifuged (1000 × g, 10 min, RT). The cell pellet was fixed in 2.5% glutaraldehyde and 1.5% formaldehyde diluted in 0.3 M 4-(2-hydroxyethyl)piperazine-1-ethanesulfonic acid buffer (HEPES, pH 7.35, Sigma-Aldrich, Merck, Cat#H4034) and stored at 4 °C according to Brinkmann et al., 2004 and Behrendt et al., 2008 (25, 71). The cell pellet was washed and post-fixed in 0.15 M HEPES buffer containing 1% osmium tetroxide. After thorough washing in distilled water, the samples were incubated overnight in 2% aqueous uranyl acetate dihydrate (Merck, Cat#8473) at RT, dehydrated in ethanol (Sigma-Aldrich, Merck, Cat#32205-M), and embedded in an epoxy resin using the Agar 100 Resin Kit (Agar Scientific, Essex, UK Cat#AGR1031). Ultrathin sections were mounted on grids and examined using a TEM Zeiss 902 (Oberkochem, Germany) at the Institute of Anatomy and Cell Biology (JLU Giessen). Brightness and contrast were processed in a standardized way using Image J software, FIJI version (70).

### Visualization of monocyte/macrophage extracellular traps via conventional and confocal immunofluorescence microscopy

1.5 x 10^5^ primary human or bovine monocytes as well as monocytic THP-1 cells or THP-1 cell-derived M0-, M1-, and M2-like macrophages were either left untreated, co-cultured for 4 h with 6 x 10^5^ *T. gondii* tachyzoites or stimulated with A23187 [5 µM] (Sigma-Aldrich, Merck, Cat#C7522) or nigericin [0.5 µM] (sodium salt, Sigma-Aldrich, Merck, Cat#N7143) on 15 mm coverslips (Carl Roth, Cat#P232.1) pre-coated with 0.01% poly-L-lysine in 12-well plates, fixed in 4% formaldehyde diluted in PBS, and stored at 4°C until further use. The fixed samples were washed three times with PBS, blocked in blocking/permeabilization solution [PBS containing 3% BSA (Sigma-Aldrich, Merck, Cat#A7906) and 0.3% Triton X-100 (Sigma-Aldrich, Merck, Cat#T8787)] for 1 h at RT and reacted with primary antibodies in blocking/permeabilization solution overnight at 4 °C. Anti-histone antibodies (clone H11-4, Sigma-Aldrich, Merck, Cat#MAB3422) and anti-myeloperoxidase (MPO) antibodies (Biorbyt, Cambridge, UK, Cat#orb16003) were used to detect corresponding proteins on extruded METs. After three washings with PBS, samples were incubated in a secondary antibody blocking/permeabilization solution (Alexa Fluor 594 goat anti-mouse IgG, Invitrogen™, Thermo Fisher Scientific, Cat#A11005) for 30 min at RT. Finally, the samples were washed three times with PBS. Coverslips were mounted on microscope slides (Glaswarenfabrik KarlHecht GmbH & Co. KG, Sondheim, Germany, Cat#42400010) with Fluoromount-G™ (Invitrogen™, Thermo Fisher Scientific, Cat#00-4959-52) to stain DNA with 4′,6-diamidino-2-phenylindole (DAPI) and incubated for 48 h at RT. To visualize METs, specimens were analyzed using an Olympus IX81 microscope (Tokyo, Japan) equipped with an Excelitas X-Cite 120PC Q fluorescence illuminator (Pittsburgh, Pennsylvania, USA) and an Olympus XM-10-R digital camera. Confocal microscopy was performed using a Nikon Eclipse Ti2-A microscope (Tokyo, Japan) equipped with a ReScan^®^ confocal unit (RCM 1.1 Visible, Confocal.nl, Amsterdam, Netherlands). The confocal microscope was equipped with a 50 μm pinhole, a Nikon motorized Z-stage (DI1500), and a Toptica CLE laser (Graefelfing, Germany) with the following excitation modes: blue/DAPI/405 nm, green/fluorescein-5-isothiocyanate (FITC)/488 nm, and red/AlexaFluor594/561 nm. An Excelitas sCMOS^®^ camera (PCO edge) was used for documentation and the instrument was operated using NIS-Elements software (version 5.11, Nikon). Z-series were displayed as maximum z-projections and gamma, brightness and contrast were processed in a standardized way using Image J software, FIJI version (70).

### Quantification of extracellular DNA

5 x 10^4^ primary human or bovine monocytes as well as 1 x 10^5^ monocytic THP-1 cells or THP-1 cell-derived M0-, M1-, and M2-like macrophages were either left untreated, co-cultured for 4 h with 2 x 10^5^ or 4 x 10^5^ *T. gondii* tachyzoites or stimulated with A23187 [5 µM] or nigericin [0.5 µM] in 96-well plates (Greiner Bio-One, Cat#655180). After incubation, Sytox Green [5 µM] (Invitrogen™, Thermo Fisher Scientific, Cat#S7020) was added to all samples to stain extDNA followed by centrifugation (400 x g, 5 min, RT). The supernatants were collected carefully to fresh 96-well plates and measured at an excitation wavelength of 488 nm using a Clariostar Plus plate reader and MARS data analysis software (version 4.20, both BMG Labtech, Ortenberg, Germany).

### Quantification of nuclear area expansion and MET positive cells

1.5 x 10^5^ primary human or bovine monocytes as well as monocytic THP-1 cells or THP-1 cell-derived M0-, M1-, and M2-like macrophages were either left untreated, co-cultured for 4 h with 6 x 10^5^ *T. gondii* tachyzoites or stimulated with A23187 [5 µM] or nigericin [0.5 µM] on 15 mm coverslips pre-coated with 0.01% poly-L-lysine in 12-well plates, fixed in 4% formaldehyde in PBS, and stored at 4 °C until further use. Fixed samples were washed three times with PBS, blocked in blocking/permeabilization solution for 1 h at RT, and washed three times with PBS. Coverslips were mounted on microscope slides with Fluoromount-G™ to stain DNA with DAPI an incubated for 48 h at RT. Cell nuclei were visualized using a Keyence BZ-X800 microscope (Osaka, Japan) and measurements were performed using the Keyence BZ-X800 analyzer software (version 1.1.2.4). The average nuclear area of DAPI-stained DNA was calculated for each cell donor/passage using a field of 5.290 x 3.968 mm. The percentage of MET positive cells was determined by analyzing 1250 cells per condition. Cells were classified as MET positive by the presence of extracellular, fibre-like DNA structures outside the cell area.

### Measurement of cytokine release via ELISA

1 x 10^5^ primary human or bovine monocytes as well as monocytic THP-1 cells or THP-1 cell-derived M0-, M1-, and M2-like macrophages were seeded into 96-well plates. Cells were either left untreated, co-cultured with 4 x 10^5^ *T. gondii* tachyzoites in the presence or absence of LPS [1 µg/ml] or treated with the known chemical activators of MET release A23187 [5 µM] or nigericin [0.5 µM] for 4 h. To proof that the used cells responded with IL-1β release to classical NLRP3 activation stimuli, cells were first primed with LPS for 3.5 h followed by exposure for 40 min to 2’/3’-O-(4-benzoylbenzoyl)adenosine-5’-triphosphate, tri(triethylammonium) salt (BzATP) [200 µM] (Jena Bioscience, Jena, Germany, Cat#NU-1620) or nigericin [25 µM] in the absence of *T. gondii* tachyzoites. Thereafter, the cells were centrifuged (500 x *g*, 8 min, 4 °C) and the cell-free supernatants were collected and stored at -20 °C for further use. IL-1β and IL-6 concentrations were measured in cell supernatants using the human IL-1 beta/IL-1F2 DuoSet enzyme-linked immunosorbent assay (ELISA; R&D Systems, Cat#DY201; assay range: 3.9 – 250 pg/ml) and the human IL-6 DuoSet ELISA (R&D Systems, Cat#DY206; assay range: 9.4 – 600 pg/ml) or the bovine IL-1 beta Uncoated ELISA Kit (Invitrogen™, Thermo Fisher Scientific, Cat#ESS0027) and the bovine IL-6 Uncoated ELISA Kit (Invitrogen™, Thermo Fisher Scientific, Cat#ESS0029).

To estimate the proportion of dead primary human or bovine monocytes as well as monocytic THP-1 cells or THP-1 cell-derived M0-, M1-, and M2-like macrophages, lactate dehydrogenase (LDH) activity was measured in the cell culture supernatants according to the supplier’s instructions of the CytoTox 96^®^ Non-Radioactive Cytotoxicity Assay (Promega, Madison, Wisconsin, USA; Cat#G1780) using a Epoch™ Microplate Spectrophotometer (BioTek Instruments, Inc., Vermont, USA). The LDH activities determined in the cell-free supernatants are expressed as a percentage of the total LDH activity of the lysed control cells.

### Detection of monocytic TLR2, TLR4, CD11b and F-actin by flow cytometry

5 x 10^5^ primary human and bovine monocytes were left in plain medium or co-cultured for 4 h with 2 x 10^6^ *T. gondii* tachyzoites in cell culture Petri dishes (Sarstedt, Cat#83.3902.500) (primary human monocytes) or in Eppendorf tubes (primary bovine monocytes), and thereafter fixed in 0.7% formaldehyde (primary human monocytes) or BD Cytofix (Franklin Lakes, New Jersey, USA, Cat#554722) (primary bovine monocytes). In case of primary human monocytes, the samples were washed in PBS containing 2% FCS and incubated for 10 min on ice with 10% heat-inactivated human serum to block Fc-receptors. Samples were washed with PBS containing 2% FCS and labelled with antibodies raised against CD11b (1:50, ImmunoTools GmbH, Friesoythe, Germany, Cat#21279116X3), TLR2 (1:10, BioLegend^®^, San Diego, USA, Cat#309708) or TLR4 (1:10, BioLegend^®^, Cat#312806) according to the manufacturer’s protocol. F-actin was detected by phalloidin coupled with Alexa 488 (abcam^®^, Cambridge, UK, Cat#ab176753). The stained cells were washed twice in PBS containing 2% FCS, centrifuged (1500 x *g*, 7 min, 4°C), resuspended in 0.5 ml PBS containing 2% FCS, and analyzed using a BD Accuri™ C6 flow cytometer (BD Biosciences, Heidelberg, Germany). In case of primary bovine monocytes, the samples were washed in PBS containing 1% BSA and labelled with antibodies raised against CD11b (BD, Cat#553312), TLR2 (Bio-Rad Laboratories, Hercules, California, USA, Cat#HCA152A647) or TLR4 (Bio-Rad Laboratories, Cat#MCA2061A647) in a dilution of 1:100 according to the manufacturer’s protocol. F-actin was detected by phalloidin coupled with Alexa 488. The stained cells were washed in PBS containing 1% BSA, centrifuged (600 x *g*, 10 min, 4°C), resuspended in 0.4 ml PBS containing 1% BSA, analyzed using a BD Accuri™ C6 flow cytometer. In the cell/parasite suspension, no discrimination between infected cells vs. uninfected cells was performed. The gating strategy and fluorescence intensity profiles for primary human and bovine monocytes are shown in **Supplementary Figure S2** and **Supplementary Figure S3**.

### Statistical analyses

Data were presented as mean ± standard deviation (SD) or median ± interquartile range (75^th^ and 25^th^ percentiles) using GraphPad Prism^®^ (version 10.2.3, GraphPad Software, Boston, Massachusetts, USA). Data analysis was performed using SAS^®^ statistical software (version 9.4, Statistical Analysis System Institute, Cary, North Carolina, USA). For all experiments, the number (n) refers to independent experiments performed on different days and different cell passages/blood donors. Outliers were not excluded from the analyses. The statistical tests used for individual data sets are indicated in the legends of the respective figures.

## Results

### Differential microscopic analyses illustrate activation of mononuclear phagocytes within 4 h of exposure to *T. gondii* tachyzoites

A panel of microscopic analyses was performed to investigate early responses of mononuclear phagocytes to *T. gondii* tachyzoites. To compare findings obtained from primary human and bovine monocytes, the human monocytic THP-1 cell line was analyzed in parallel.

**Figure 1:**
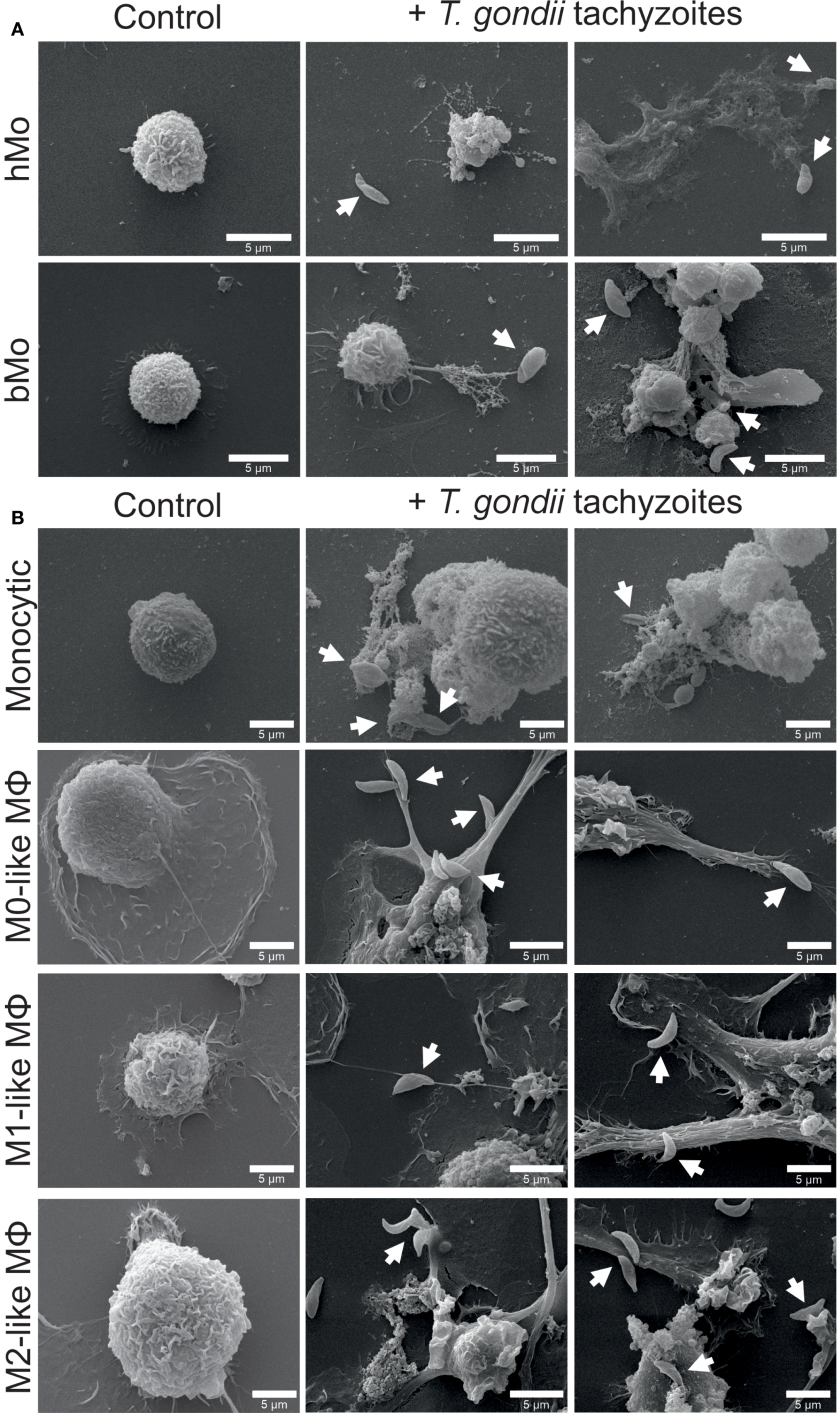
Exposure to *Toxoplasma gondii* tachyzoites triggers phenotypic changes in primary human and bovine monocytes, monocytic THP-1 cells and THP-1 cell-derived macrophages. Untreated (control) and parasite-exposed (+ *T. gondii* tachyzoites) primary human (hMo) and bovine monocytes (bMo) **(A)**, monocytic THP-1 cells (monocytic) as well as THP-1 cell-derived M0-, M1-, and M2-like macrophages (MΦ) **(B)** were fixed after 4 h of co-incubation with *T. gondii tachyzoites* (MOI 1:4) and analyzed by scanning electron microscopy (SEM). Interactions of cells with *T. gondii* tachyzoites are indicated by white arrows. Representative pictures from n = 3 independent experiments are shown.

In SEM analyses, primary untreated (control) human and bovine monocytes revealed typical spherical shapes and surfaces with little cytoplasmic spreading and ruffling (26, 72) (**Figure 1A**).

SEM images show that both primary human and bovine monocytes exhibit morphological changes (e.g. protrusion) upon exposure to the parasite, similar to those observed in other mononuclear phagocytes (**Figure 1A**, **Supplementary Figure S4**) (73–75). Compared with non-stimulated primary human and bovine monocytes, *T. gondii* tachyzoites induced cytoplasmic spreading, cell surface ruffling, formation of membrane protrusions by filopodia and cytonemes, and MET formation. The release of METs was accompanied by an extrusion of a large amount of cell debris as well as parasite entrapment, thereby probably corresponding to ‘suicidal METosis’ (**Figure 1A**). Untreated monocytic THP-1 cells presented a round-shaped and loosely attached phenotype, while THP-1 cell-derived macrophages had an irregular expanded surface and showed pronounced adhesion capacity (**Figure 1B**). Exposure to *T. gondii* tachyzoites induced membrane protrusions, mainly consisting of filopodia (**Figure 1B**). Similar results were found in Pappenheim-stained THP-1 cell-derived macrophages (**Supplementary Figure S5**).

**Figure 2:**
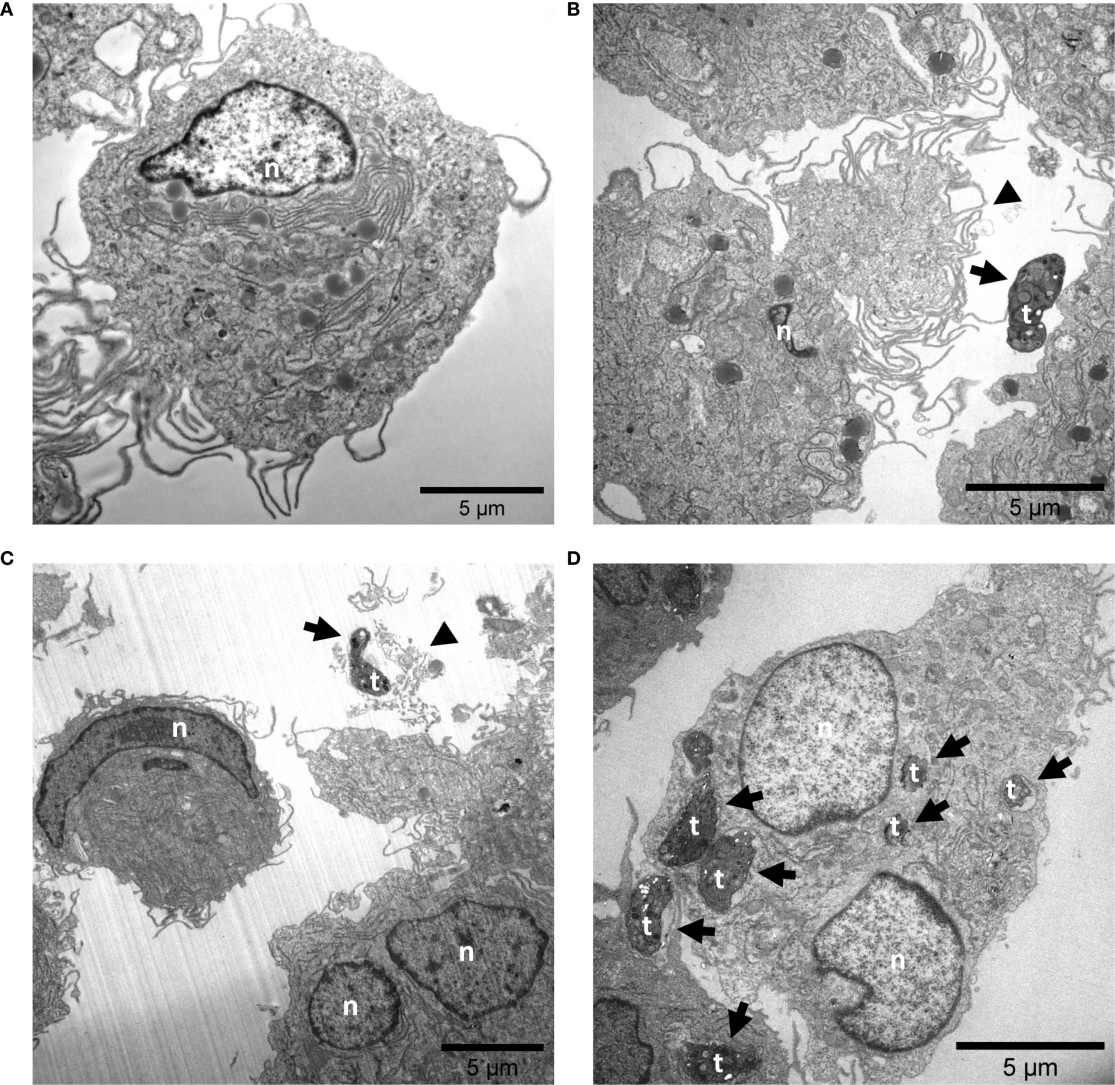
Exposure to *Toxoplasma gondii* tachyzoite causes the release of ET-like structures by M1-like macrophages. Untreated **(A)** and *T. gondii* tachyzoite-exposed M1-like macrophages (MOI 1:4) **(B-D)** were fixed after 4 h of co-incubation and analyzed by transmission electron microscopy (TEM). The black arrowheads points at regions that might correspond to extracellular trap release from M1-like macrophages **(B, C)**. In addition, several *T. gondii* tachyzoites were observed in intracellular position **(D)**. Interactions of M1-like macrophages with *T. gondii* tachyzoites are indicated by black arrows. n, nuclear; t, tachyzoite.

In addition, TEM analyses were performed and confirmed the typical ultrastructure of THP-1 cell-derived macrophages (**Figure 2**) (76). THP-1 cell-derived M1-like control macrophages exhibited an ameboid shape and a large number of cytoplasmic granules, vesicles and vacuoles (**Figure 2A**). *T. gondii* exposure induced cell protrusions (**Figure 2B**). Moreover, ET-like structures, which contained and eventually trapped *T. gondii* tachyzoites, were observed in juxtaposition to M1-like macrophages (**Figures 2B, C**). Besides, several intracellular *T. gondii* tachyzoites were found, partially enclosed by a distinct membrane, resembling that of phagolysosomes or parasitophorous vacuoles (PV) (**Figure 2D**).

**Figure 3:**
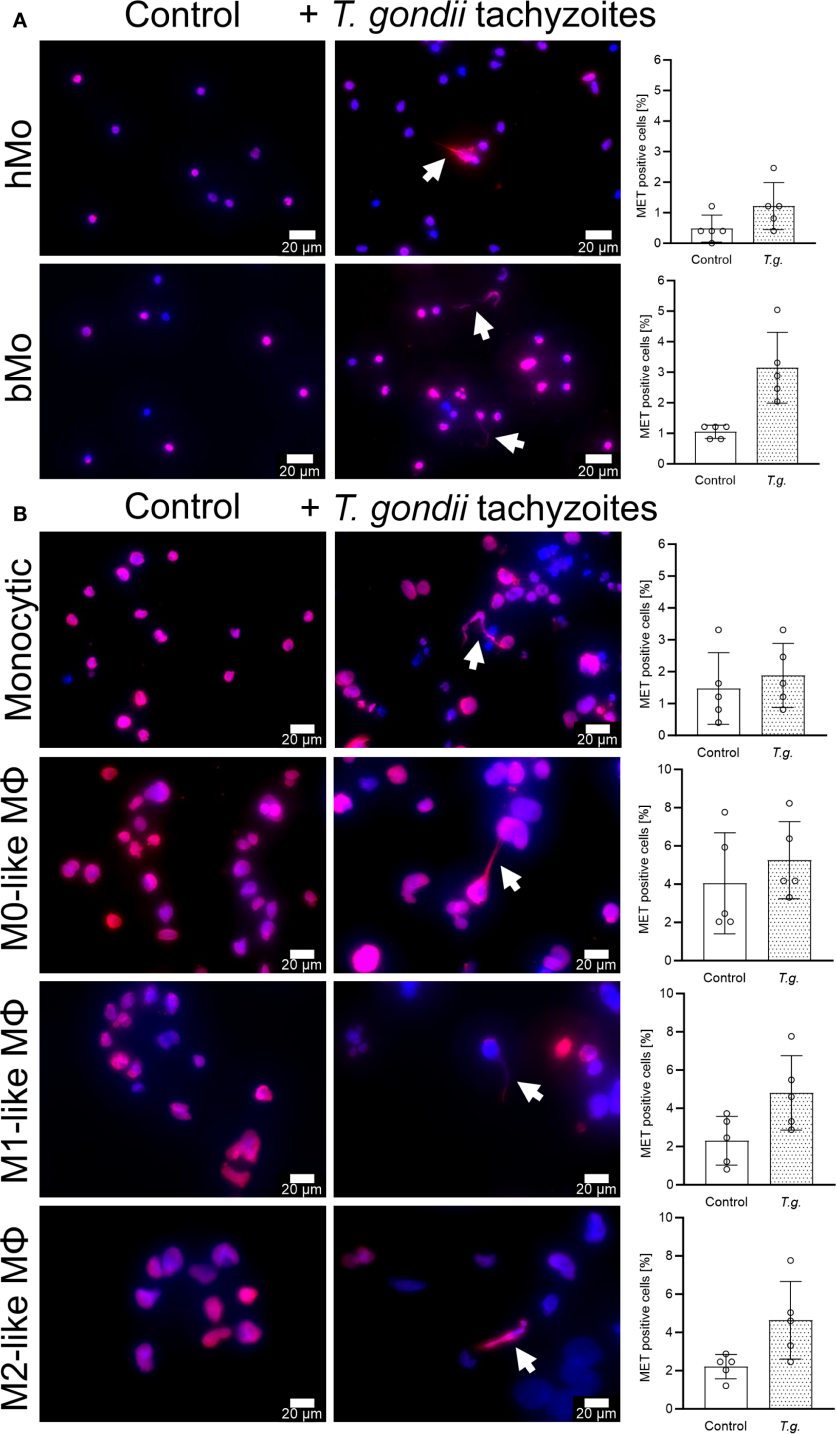
Extracellular trap marker detection in co-cultures of *Toxoplasma gondii* tachyzoites with primary human and bovine monocytes, monocytic THP-1 cells and THP-1 cell-derived macrophages. Primary human (hMo) and bovine monocytes (bMo) **(A)**, monocytic THP-1 cells (monocytic) as well as THP-1 cell-derived M0-, M1-, and M2-like macrophages (MΦ) **(B)** were left untreated (control) or exposed to *T. gondii tachyzoites* (+ *T. gondii* tachyzoites, *T*.*g*., MOI 1:4). The cells were fixed after 4 h of incubation and stained for DNA (blue) and histone (red). Extracellular traps, which are characterized by the co-localization of DNA and histones, are indicated by white arrows. Percentage of monocyte/macrophage extracellular trap (MET) positive cells was determined (n = 5). The graphs show mean with standard deviation (SD). Statistical analysis was performed by comparing *T. gondii*-exposed cells with controls using the non-parametric Wilcoxon signed-rank test; no p-values below p ≤ 0.05 were observed.

Immunofluorescence microscopy illustrated filamentous structures showing the co-localization of extDNA with classical ET markers like histone and MPO and thereby confirmed METs being released in response to *T. gondii* tachyzoites from all, primary human and bovine monocytes, monocytic THP-1 cells, and THP-1 cell-derived macrophages (**Figures 3A, B**; **Supplementary Figure S6** **;** **Supplementary Figures S7A, B**). When quantifying % of MET positive cells, only minor tachyzoite-driven effects were detected (**Figures 3A, B**). The weak or lack of a significant induction of parasite-driven METs indicated that MET release from primary human and bovine monocytes as well as all kinds of THP-1 cells, was a rare event (**Figures 3A, B**). Similar results were obtained by using the MET inducers A23187 (5 µM, 4 h) and nigericin (0.5 µM, 4 h) (**Supplementary Figures S8A, B**) (20, 36).

**Figure 4:**
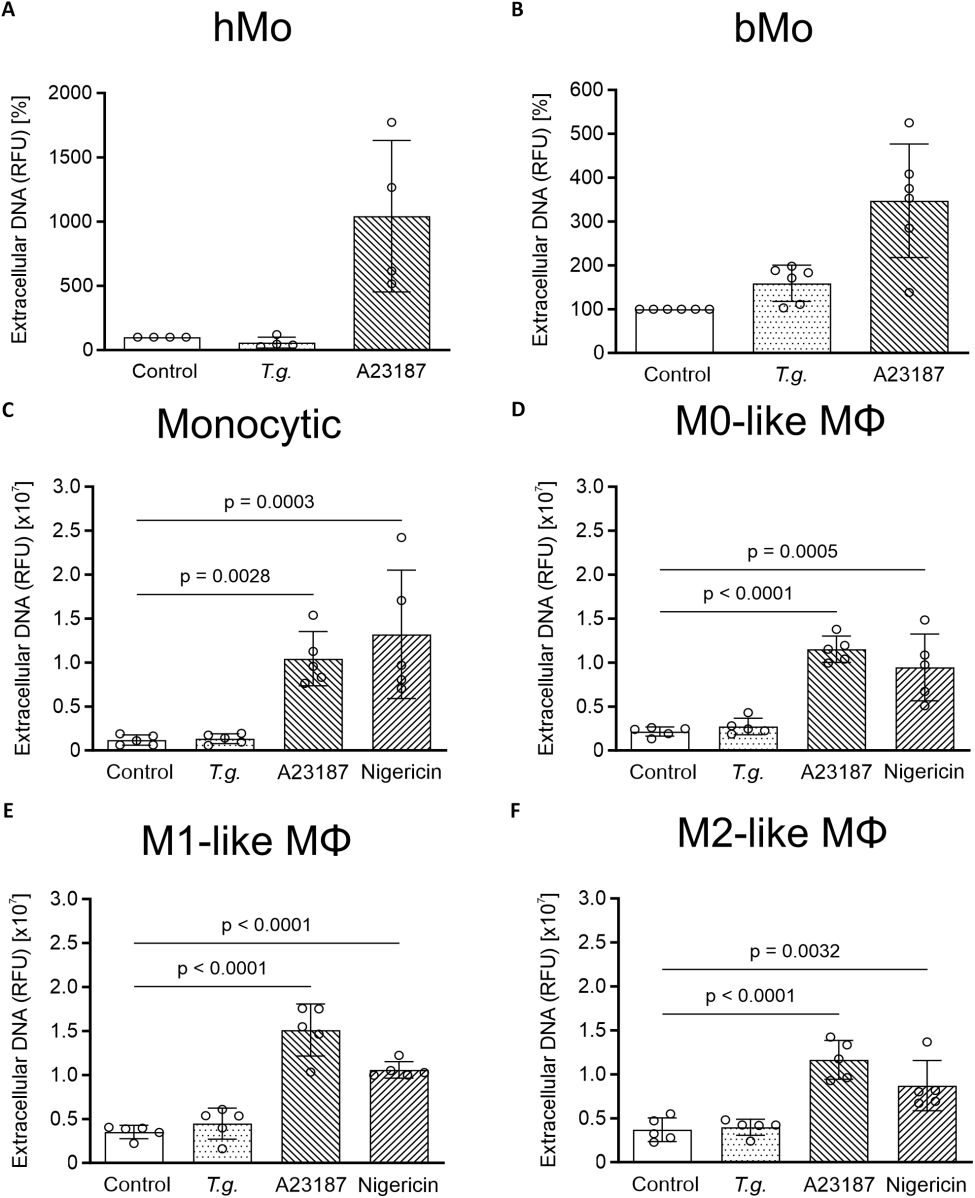
Extracellular DNA in supernatants from *Toxoplasma gondii*-exposed primary human and bovine monocytes, monocytic THP- cells and THP-1 cell-derived macrophages. Supernatants of untreated (control) primary human (hMo) **(A)** and bovine monocytes (bMo) **(B)**, monocytic THP-1 cells (monocytic) **(C)** as well as THP-1 cell-derived M0- **(D)**, M1- **(E)**, and M2-like macrophages (MΦ) **(F)**, *T. gondii*-exposed cells (*T*.*g*., MOI 1:4) or treated cells with A23187 [5 µM] or nigericin [0.5 µM] were collected after 4 h of incubation. Sytox Green was added to the supernatants, followed by spectrophotometric analysis. The amount of extracellular DNA is indicated as relative fluorescence units (RFU) (n = 4 – 6). For both hMo and bMo, controls were normalized to 100% and treatment values were adjusted accordingly to account for inter-donor differences. The graphs show mean with standard deviation (SD) and p-values below 0.05 (p ≤ 0.05). Statistical analysis was performed by comparing *T.g.*-exposed, A23187- or nigericin-treated cells for primary monocytes using the Friedman test, followed by pairwise comparisons via the Wilcoxon signed-rank test, followed by the Bonferroni correction *post-hoc* pairwise comparison, and for all kinds of THP-1 cells using a two-way analysis of variance (ANOVA) with residual normality assessed to validate model assumptions, followed by the Bonferroni correction *post-hoc* pairwise comparison.

In line with the fluorescence microscopic data, neither nuclear area expansion (NAE) estimation (**Supplementary Figure S9**) nor fluorometric determination and quantification of extDNA (**Figure 4**) showed a tachyzoite-driven upregulation of METosis in primary human and bovine monocytes, monocytic THP-1 cells, and THP-1 cell-derived macrophages. As expected, stimulation with the positive controls A23187 [5 µM] and nigericin [0.5 µM] (20, 36) significantly induced extDNA release by monocytic THP-1 cells and THP-1 cell-derived macrophages (p ≤ 0.05; n = 5; **Figures 4C-F**).

### Effects of *T. gondii* tachyzoite exposure on release of IL-1β and IL-6

**Figure 5:**
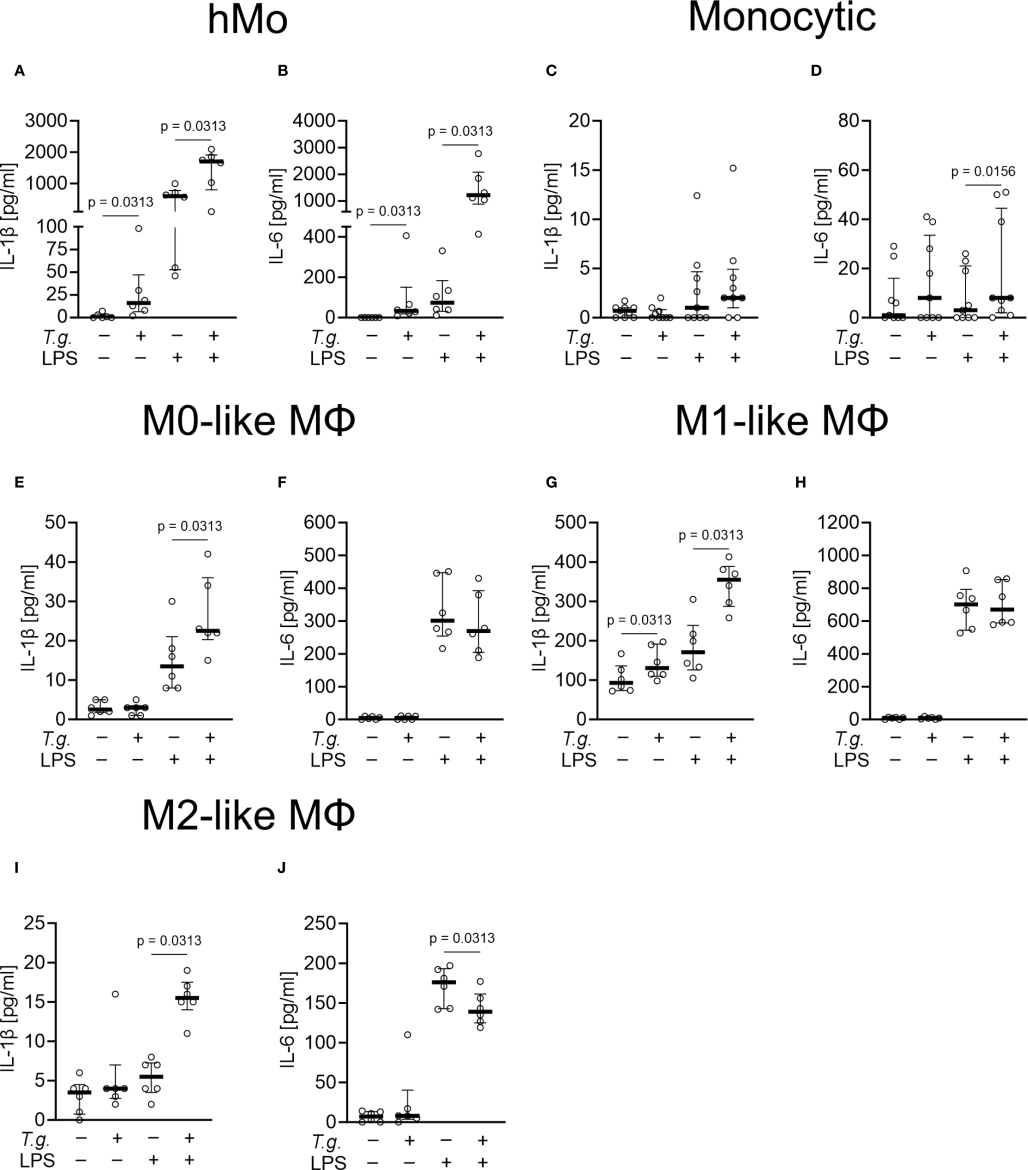
Release of interleukin (IL)-1β and IL-6 by *Toxoplasma gondii* tachyzoite-exposed primary human monocytes, monocytic THP-1 cells and THP-1 cell-derived macrophages. Primary human monocytes (hMo) **(A, B)**, monocytic THP-1 cells (monocytic) **(C, D)** as well as THP-1 cell-derived M0- **(E, F)**, M1- **(G, H)**, and M2-like macrophages (MΦ) **(I, J)** were left untreated (control), exposed to *T. gondii* tachyzoites (*T*.*g*., MOI 1:4) in the absence or presence of LPS [1 µg/ml] or primed with LPS [1 µg/ml] alone for 4 h each (n = 6 – 9). The concentrations of IL-1β **(A, C, E, G ,I)** and IL-6 **(B, D, F, H, J)** were measured in cell culture supernatants by ELISA. The graphs show median with the interquartile range (75^th^ and 25^th^ percentiles) and p-values below 0.05 (p ≤ 0.05). Statistical analysis was performed by comparing *T.g*.-exposed cells with controls or *T.g*.-exposed/LPS-treated cells with LPS-treated cells using the Friedman test, followed by pairwise comparisons via the Wilcoxon signed-rank test.

Activation of mononuclear phagocytes by pathogens can lead to the release of pro-inflammatory cytokines (13). Here, we investigated if the early response of monocytes and macrophages involves secretion of pro-inflammatory cytokines. To determine the release of the pro-inflammatory cytokines IL-1β and IL-6 by primary human monocytes, monocytic THP-1 cells, and THP-1 cell-derived M0-, M1-, and M2-like macrophages were exposed to *T. gondii* tachyzoites in absence or presence of LPS (**Figure 5**). We here focused on human mononuclear phagocytes, since commercial ELISAs for the bovine system did not produce plausible results and were therefore discarded (data not shown). Untreated primary human monocytes released low amounts of IL-1β ranging from < 3.9 to 7 pg/ml and IL-6 < 9.4 pg/ml (n = 6) (**Figures 5A, B**). Similar results were found for untreated monocytic THP-1 cells with IL-1β levels from < 3.9 pg/ml and IL-6 from < 9.4 to 29 pg/ml (n = 9) (**Figures 5C, D**). *T. gondii* tachyzoites enhanced the release of IL-1β (from < 3.9 to 98 pg/ml) and of IL-6 (from < 9.4 to 406 pg/ml) by primary human monocytes (p ≤ 0.05; n = 6) (**Figures 5A, B**) but had no effect on IL-1β and IL-6 secretion by monocytic THP-1 cells (**Figures 5C, D**). Only in M1-like macrophages, exposure to *T. gondii* tachyzoites enhanced the release of IL-1β ranging from 13 to 125 pg/ml (p ≤ 0.05; n = 6) but had no effect on IL-6 release (**Figures 5G, H**).

Interestingly, exposure to *T. gondii* tachyzoites induced additive effects in LPS-primed cells. While LPS-primed primary human monocytes released IL-1β ranging from 46 to 996 pg/ml and IL-6 ranging from 11 to 331 pg/ml, *T. gondii* tachyzoites exposure increased IL-1β release (from 105 to 2087 pg/ml) and IL-6 (from 413 to 2772 pg/ml) (p ≤ 0.05; n = 6) (**Figures 5A, B**). Similarly, the LPS-induced release of IL-1β by THP-1 cell-derived macrophages (M0-like: ranging from 8 to 30 pg/ml; M1-like: ranging from 105 to 305 pg/ml; M2-like: ranging from < 3.9 to 8 pg/ml) was increased by tachyzoites exposure with the highest levels detected in supernatants of M1-like macrophages (ranging from 156 to 329 pg/ml; p ≤ 0.05; n = 6) (**Figures 5E, G, I**). Interestingly, no impact of *T. gondii* tachyzoites exposure was detected on LPS-induced IL-6 release by M0- and M1-like macrophages (**Figures 5F, H**). A moderate increase of IL-6 release was detected in LPS-primed monocytic THP-1 cells (ranging from < 9.4 to 26 pg/ml; p ≤ 0.05; n = 9) (**Figure 5D**), whereas a diminished IL-6 secretion was detected in LPS-primed M2-like macrophages (p ≤ 0.05; n = 6) (**Figure 5J**). Of note, the parasite-driven enhancement of IL-1β release was not due to monocyte or macrophage cell death since LDH values remained low (< 10% increase) in all experimental settings (n = 6 – 9) **Supplementary Table S1**).

To prove that the used cells responded with IL-1β release to classical NLRP3 activation, stimuli BzATP [200 µM] or nigericin [25 µM] were used as positive controls. For this purpose, the cells were first primed with LPS for 3.5 h followed by exposure for 40 min to BzATP [200 µM] or nigericin [25 µM]. As expected (77), all tested human mononuclear phagocytes responded with an increased release of IL-1β with the highest levels detected in supernatants of M1-like macrophages (ranging from 427.0 to 2899.0 pg/ml; n = 6) (**Supplementary Table S2**). In addition, we tested the effects of the MET inducers A23187 [1 µM] and nigericin [0.5 µM] after 4 h of incubation (**Supplementary Table S2**). Of note, high values of cell death were estimated in the presence of A23187 and nigericin with a maximum of 80% cell death in M1-like macrophages treated with A23187 (**Supplementary Table S2**).

Taken together, the current data suggest that exposure to *T. gondii* tachyzoites activates primary human monocytes and fosters the release of pro-inflammatory cytokines. We found subtype-specific differences in the THP-1 cells, with THP-1 cell-derived M0- and M1-like macrophages as responding cells to *T. gondii* tachyzoites exposure.

### Tachyzoite exposure does not affect TLR2, TLR4, F-actin, and CD11b expression in primary human and bovine monocytes

It was shown before, that TLR2 and TLR4 expression on bovine neutrophils increased when confronted with the apicomplexan parasite *Eimeria bovis* (78). In addition, *T. gondii*-derived GPI proteins showed TNF-α production dependent on TLR2 and TLR4 on mice macrophages (79). Thus, we here evaluated the changes in TLR2 and TLR4 in primary human- and bovine monocytes exposed to *T. gondii* tachyzoites for 4 h. The results showed no significant differences (p ≥ 0.05, n = 4 – 6) when compared with the unexposed controls (**Supplementary Figure S10**). In addition, the cellular membrane expression of CD11b and F-actin polymerization, known factors involved in parasite phagocytosis and immune cell migration was analyzed (80). In the context of primary human and bovine monocyte activation by *T. gondii* exposure no significant changes were observed between the unexposed and the *T. gondii*-exposed cells (p ≥ 0.05; n = 4 – 6) (**Supplementary Figure S10**).

## Discussion

In the present study, we focused on early immune responses of human and bovine mononuclear phagocytes against vital *T. gondii* tachyzoites on the level of morphological changes, MET release, pro-inflammatory cytokine secretion and surface receptor expression. In addition to freshly isolated primary human and bovine monocytes, we used monocytic THP-1 cells and THP-1 cell-derived macrophages, that are widely used in research and represent well-accepted *in vitro* models for human mononuclear phagocytes (30, 32). Despite the critical role of monocytes and macrophages in early innate reactions, investigation on monocyte-derived immune response against protozoan and metazoan parasites is still an understudied topic in both human and veterinary medicine. Thus, their role in the context of toxoplasmosis remains unclear (81). Of note, one report on *T. gondii* tachyzoite-driven MET formation in harbour seal (*Phoca vitulina*) exists (18).

Exposure to *T. gondii* tachyzoites induced morphological changes in investigated monocytes and macrophages, including cytoplasmic spreading, cell surface ruffling, and the formation of membrane protrusions by filopodia and cytonemes, as well as MET formation.

These findings are in line with previous research showing that *T. gondii* infection triggers significant morphological and functional changes in various mononuclear phagocytes. Across different cell types, including dendritic cells, microglia, and macrophages, tachyzoite invasion consistently induces rapid cytoskeletal remodeling, characterized by podosome loss, cytoplasmic spreading, and the formation of membrane protrusions, which collectively contribute to a hypermigratory phenotype (73–75). The parasite effector *Tg*WIP has been identified as a central mediator of these processes by modulating host cell migration (82), suggesting a conserved strategy by which *T. gondii* manipulates host immune cell behavior.

Like NETosis (83), the ability of primary monocytes to release METs in response to a variety of stimuli is a well-established effector mechanism (18–21, 23, 24, 26, 27, 29). As key players in the immune defense against toxoplasmosis, macrophages influence the host’s ability to control the infection through their functional polarization (54, 84, 85). MET formation was already reported for monocytic THP-1 cells and THP-1 cell-derived M0-, and M1-like macrophages in response to a range of different stimuli e.g. chemicals, parasite and bacteria (17, 34–41). This is in principle in line with our findings in microscopic analyses (SEM, TEM, and Pappenheim-staining), where amorphous extracellular material was associated with *T. gondii* tachyzoites exposure, eventually corresponding to MET formation. However, the nuclear membranes of *T. gondii*-exposed M1-like macrophages remained intact, suggesting that MET formation did not occur in the majority of these cells (86, 87). THP-1 cell-derived macrophages are capable of phagocytosis (30, 88–91) and we found beside extracellular presence, several tachyzoites also intracellularly in M1-like macrophages. However, our analyses cannot distinguish between phagocytosis and active tachyzoite infection. In future studies heat-inactivated *T. gondii* tachyzoites could be useful to further clarify this.

To unambiguously identify MET formation, we stained extracellular structures in immune cell-parasite co-cultures for classical ET markers e.g. DNA, histones and MPO. For positive controls, the ET-inducing stimulants A23187 and nigericin (20, 36) were used. Immunostaining experiments confirmed the presence of MET positive cells but in contrast to previous findings (20), this was an extremely rare event, even when the known MET inducers A23187 or nigericin were used as positive controls. By using another established spectrofluorometric method for measuring extDNA in cell culture supernatants (92), exposure to *T. gondii* showed no changes in all tested cells. Furthermore, A23187 treatment resulted in elevated levels of cell death, as indicated by increased LDH activity in the corresponding cell-free supernatants. Consequently, it is not possible to clearly distinguish between the effects attributable to MET formation and those resulting from cell death. In this context, Matsubara et al., 1994 have shown in six human leukemia cell lines that exposure to A23187 [1 μM] induced cell apoptosis within 3 h (93), indicating that at least A23187 should be used as a positive control for MET formation with caution. We and others (20, 24, 36) used even higher concentrations and a longer exposure time. To further substantiate our findings, we next measured NAE, an established method to estimate the very early phase of ETosis (61, 94). Again, neither *T. gondii* exposure nor stimulation with A23187 or nigericin enhanced NAE and, thus, no significant induction of METosis was quantifiable.

In summary, our findings indicate that MET formation is a rather rare event and therefore most probably of minor importance in the early response of mononuclear phagocytes to *T. gondii* tachyzoites. Obviously, these data underline immune cell type-specific responses, since *T. gondii* tachyzoites were recently proven as potent ET inducers in primary neutrophils of human, bovine, murine, cat, dog, sheep, goat, bottlenose dolphin, and harbour seal origin (18, 87, 95–99).

As mentioned above, the role of IL-1β and IL-6 in *T. gondii*-driven immune responses is controversial. In previous reports, the exposure of primary human monocytes to *T. gondii* tachyzoites after 4, 16 and 24 h induced the release of IL-1β (53, 55, 57, 58) but not of IL-6 (59). *T. gondii* tachyzoite-induced release of IL-1β (53, 55, 64) or IL-6 (62, 63) after 4, 8, 12, 24 h by monocytic THP-1 cells and THP-1 cell-derived M0-like macrophages was consistently demonstrated.

To gain further insights into monocyte- and macrophage-mediated immune responses during early *T. gondii* infection, we next examined the release of IL-1β and IL-6. In addition to primary human monocytes, we performed experiments on the THP-1 cell line, allowing controlled culture and treatment conditions. In line with previous studies (53, 55, 62–64), we found that exposure to *T. gondii* tachyzoites activates human mononuclear phagocytes and fosters the release of pro-inflammatory cytokines. Importantly, we found differences within the THP-1 cell subtypes. The THP-1 cell-derived M1-like macrophages reacted by an increased release of IL-1β following exposure to *T. gondii*, and this effect was enhanced by addition of LPS. These data might indicate that parasite phagocytosis may result in a more pronounced cytokine response in M1-like macrophages, which is in accordance to previous studies (100, 101). In LPS-primed M2-like macrophages, a subtle but statistically significant decrease in IL-6 release was observed. A similar effect was described before for LPS-primed M0-like macrophages after stimulation with *T. gondii* lysate (102). Moreover, Salazar Gonzalez et al., 2014 showed that *T. gondii* profilin induced the release of IL-6 by primary human monocytes (61). A possible explanation for the contrasting findings could be the different experiment conditions (e.g. exposure times and MOI) used in different studies.

In line with previous studies (64, 98), here, we investigated the early responses of mononuclear phagocytes after 4 h of parasite exposure applying MOI of 1:4. Other studies observed an increased release of IL-1β by monocytic THP-1 cells after 12 h (MOI 1:2) and 18 h (MOI 1:3) (53, 55) or by M0-like macrophages after 4 h (MOI 1:10) (64) exposure to the parasite. The use of different parasite strains is not expected to account for these differences, as the same *T. gondii* RH strain was used (55, 64). Investigating higher MOIs in combination with variable exposure times could, however, in future studies provide more comprehensive insight into innate host responses. As mentioned before, the release of IL-1β typically requires a priming phase (to induce pro-IL-1β expression) followed by a second stimulus (DAMPs, PAMPs) for cleavage and release of the mature IL-1β (42–45). This could be an explanation for the observed enhanced IL-1β release in LPS-primed M1-like macrophages. Studies using longer exposure times (53, 55) or higher infection rates (64) might favor the release of other DAMPs, which eventually results in activated IL-1β secretion. Contamination of mononuclear phagocytes with LPS or damage-induced priming during the isolation process of primary cells might also pre-activate mononuclear phagocytes (103) and, thus, result in differences in their sensitivity to *T. gondii* tachyzoites. To validate the immunological activity of mononuclear phagocytes, we here included control experiments that assessed their responsiveness to well-characterized stimuli. By priming cells with LPS and subsequently treating them with a second stimulus (BzATP or nigericin), we induced cytokine secretion and evaluated the functional capacity of the cells. One of the key control experiments involved the use of the P2X7 receptor agonist BzATP and the pore-forming reagent nigericin to induce NLRP3 inflammasome activation. The NLRP3 inflammasome is a multi-protein complex that plays a crucial role in the innate immune response by promoting the maturation and secretion of pro-inflammatory cytokines, such as IL-1β (104). Activation of the NLRP3 inflammasome requires two signals: a priming signal, typically provided by PRRs agonists like LPS, and a second signal, which can be delivered by various DAMPs or PAMPs (42–45). BzATP, a stable adenosine triphosphate (ATP) analog, acts as an agonist for the P2X7 receptor, which is known to mediate NLRP3 inflammasome activation through potassium efflux (105). Nigericin, on the other hand, is a potassium ionophore that disrupts intracellular potassium homeostasis, leading to NLRP3 inflammasome assembly and activation (106). In current control experiments, both BzATP and nigericin reliably induced NLRP3 inflammasome-dependent IL-1β release, demonstrating the functional integrity of the mononuclear phagocytes used in our study. The successful induction of IL-1β secretion by LPS and BzATP thereby confirmed the immunological activity of the cells and the validity of the current experimental approach. Furthermore, in all experiments LDH levels were determined to test for cell death.

TLR2 and TLR4 were both shown to be involved in the immune response against *T. gondii.* While TLR2 was shown to recognize the parasitic surface profilin or GPI proteins and stimulating the production of IL-12, TLR4 rather plays a supportive role, especially at high infectious doses, as summarized for several mouse models (47, 48, 79). We further determined the expression levels of F-actin and CD11b. F-actin is part of the cytoskeleton and known to be involved in phagocytosis (106). CD11b plays a critical role in the migration and adhesion of immune cells, including monocytes (80). In *T. gondii*-infected primary human monocytes, a significant increase in the expression of CD11b in the high-affinity form of macrophage-1 antigen (Mac-1) was observed, which promotes increased crawling distance and transmigration across endothelial barriers, thus supporting the spread of the parasite in the host (80). In the current study, we did, however, not find any effects of *T. gondii* tachyzoite exposure on TLR2, TLR4, F-actin or CD11b expression by primary human and bovine monocytes. However, the lack of distinction between infected and uninfected cells complicates the interpretation of surface marker expression and represents a limitation of this study.

Taken together, we here characterized the early innate immune responses of human and bovine mononuclear phagocytes to vital *T. gondii* tachyzoites, providing valuable insights into the initial interactions between this zoonotic parasite and professional phagocytes. Our findings demonstrate that exposure to *T. gondii* tachyzoites very early activates monocytes and macrophages, leading to the release of pro-inflammatory cytokines IL-1β and IL-6, particularly when co-stimulated with LPS. This cytokine response appeared more important than the formation of METs, which was observed as a rare event. The absence of significant changes in the expression of key markers (TLR2, TLR4, F-actin, and CD11b) by primary monocytes upon exposure to *T. gondii* suggests that the early immune response is primarily mediated through cytokine secretion rather than by changes on cell surface receptor expression or cytoskeletal rearrangements. These results highlight the importance of cytokine signaling in the early innate immune response to *T. gondii* and provide a foundation for further investigations into the molecular mechanisms underlying this response. Future studies should focus on elucidating the signaling pathways involved in cytokine production and exploring the potential therapeutic targeting of these pathways to modulate the immune response against *T. gondii* infection. Further studies using standardized methods are needed to clarify the role of METs in advanced toxoplasmosis and their possible contribution to immune-related tissue damage *in vivo*, as reported for NET extrusion.

## Data Availability

The raw data supporting the conclusions of this article will be made available by the authors, without undue reservation.
