# Supplementary material for: Early responses of primary human and bovine monocytes, monocytic THP-1 cells and THP-1 cell-derived macrophages to vital Toxoplasma gondii tachyzoites
Source: Front Immunol. 2025 Oct 30;16:1683634. doi: 10.3389/fimmu.2025.1683634 (PMC12611918; doi:10.3389/fimmu.2025.1683634)
Supplement: Supplementary file 1 [file DataSheet1.pdf]

Supplementary Material

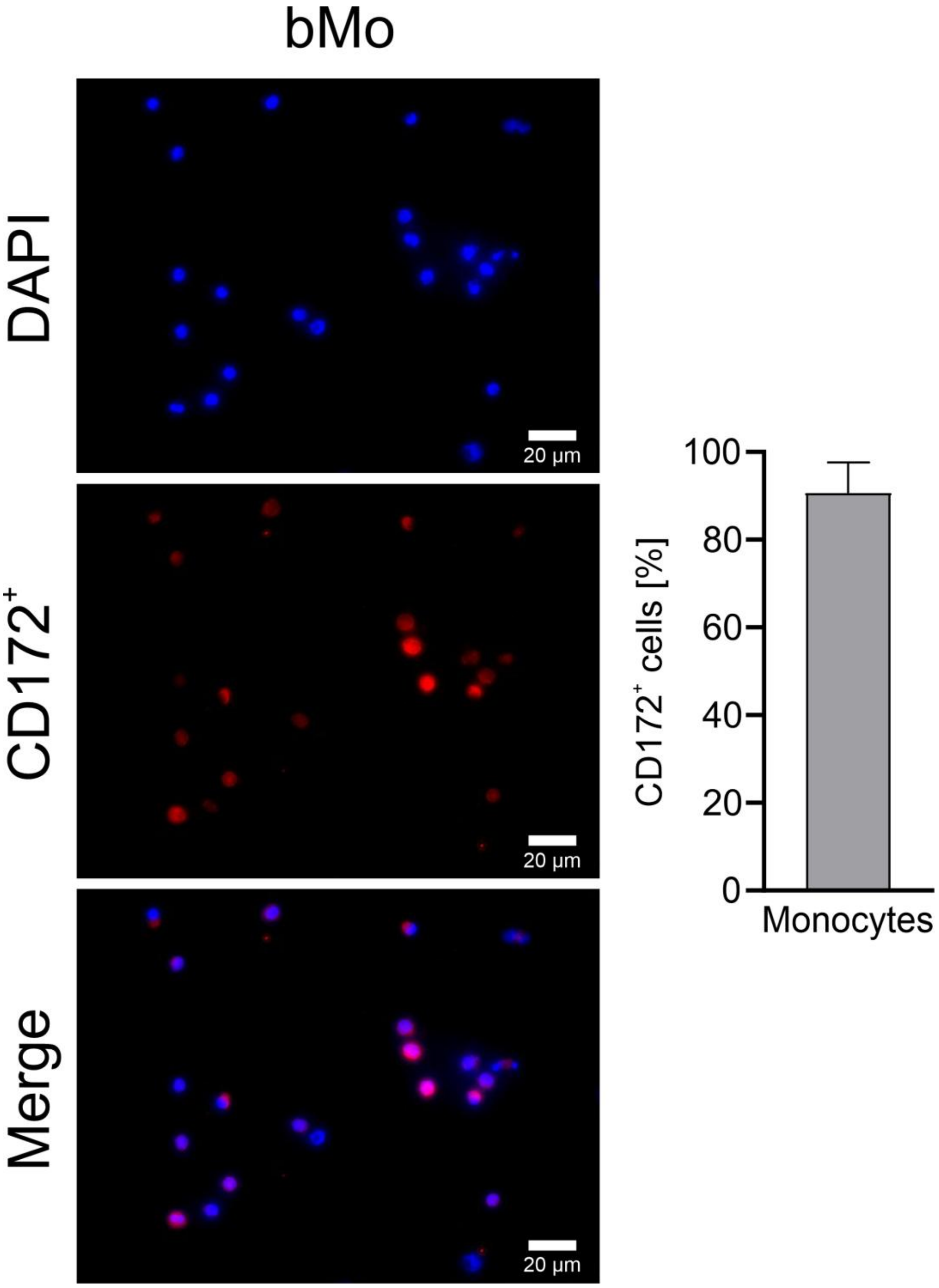

Supplementary Figure S1: Purity of isolated primary bovine monocytes.

Primary bovine monocytes (bMo) were fixed after adherence selection and stained for DNA (DAPI, blue) and with fluorescently labelled antibody to $CD172a^+$ (red). DNA-stained and $CD172a^+$ cells were counted (n = 3 animals) and expressed as the percentage of all cells. The graph shows the mean with standard deviation (SD).

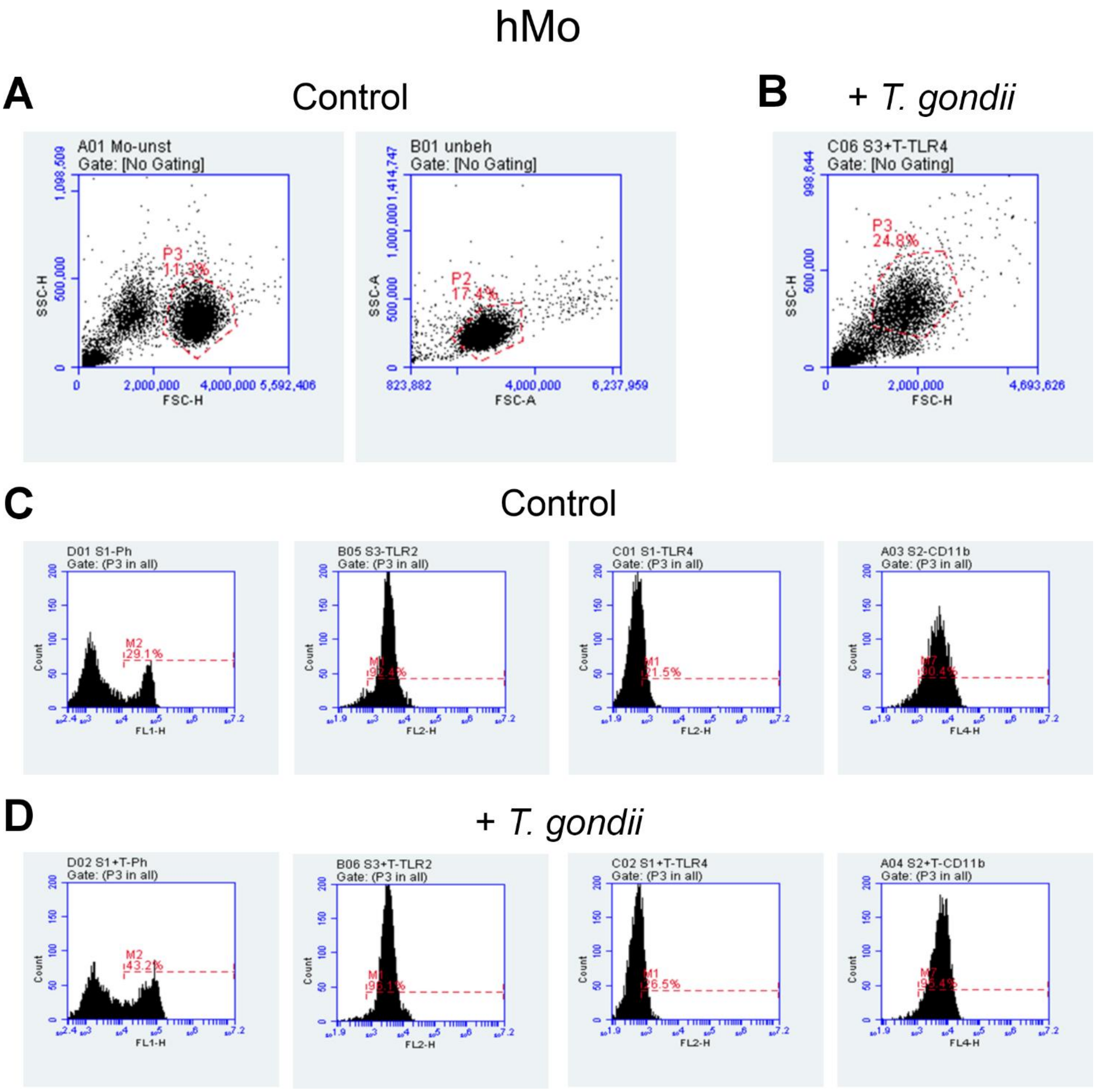

Supplementary Figure S2: Detailed gating strategy and fluorescence intensity for flow cytometry analysis of primary human monocytes (hMo).

Representative images of the gating strategy for primary hMo pre- and post-selection using immunomagnetic human CD14^{+} microbeads in untreated (control) (**A**) and *T. gondii* tachyzoite-exposed (*T. gondii*, MOI 1:4) cells **(B)**. The lower panels show representative images of the fluorescence intensities of the used markers (F-actin (phalloidin, PH), Toll-like receptor (TLR) 2, TLR4 and CD11b) in control (**C**) and *T. gondii*-exposed cells (**D**). These that complement **Supplementary Figure S10**.

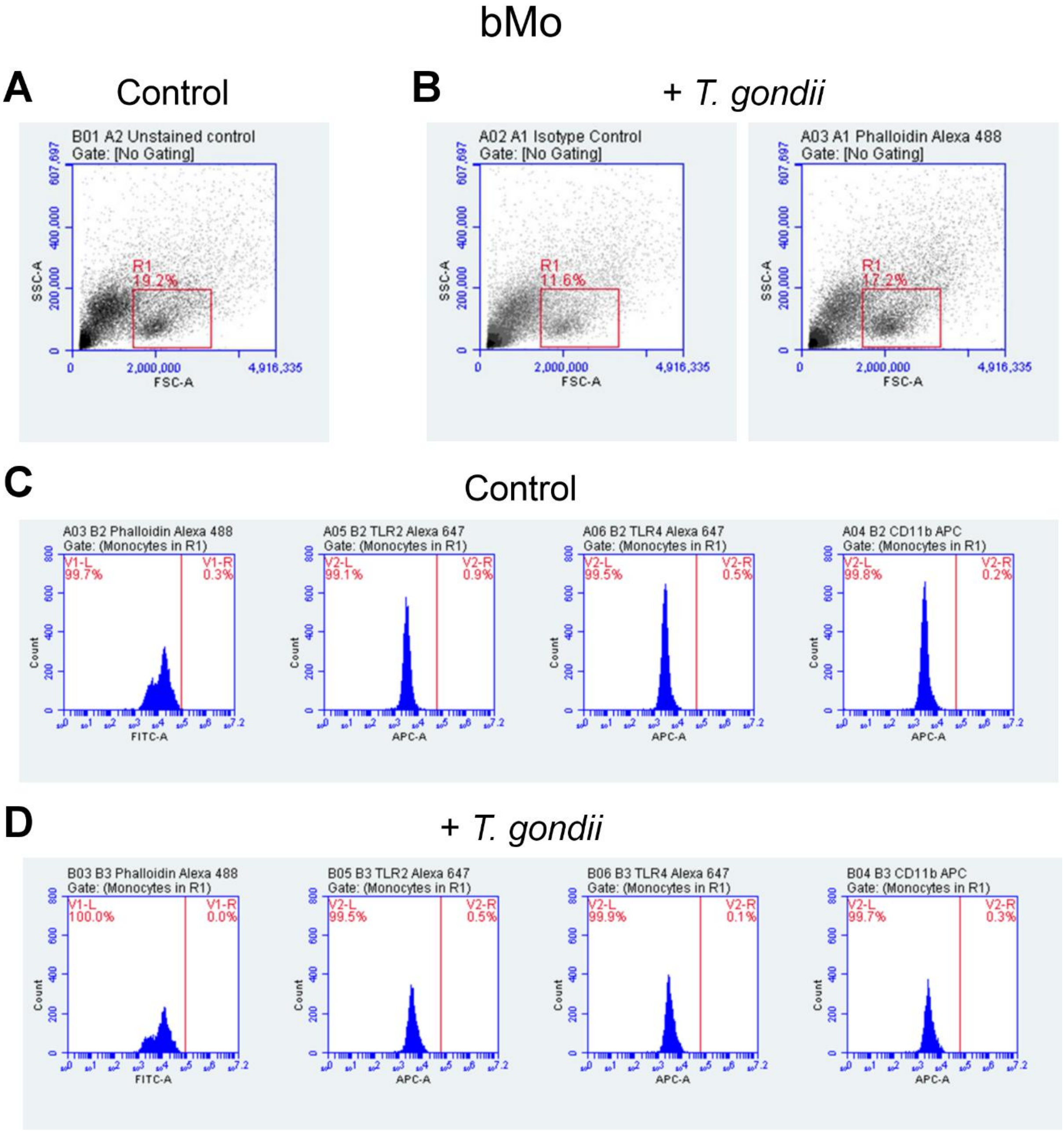

Supplementary Figure S3: Detailed gating strategy and fluorescence intensity for flow cytometry analysis of primary bovine monocytes (bMo).

Representative images of the gating strategy for primary bMo in untreated (control) (**A**) and *T. gondii* tachyzoite-exposed (*T. gondii*, MOI 1:4) cells **(B)**. The lower panels show representative images of the fluorescence intensities of the used markers F-actin (phalloidin), Toll-like receptor (TLR) 2, TLR4 and CD11b in untreated (**C**) and *T. gondii*-exposed cells (**D**). These data complement **Supplementary Figure S10.**

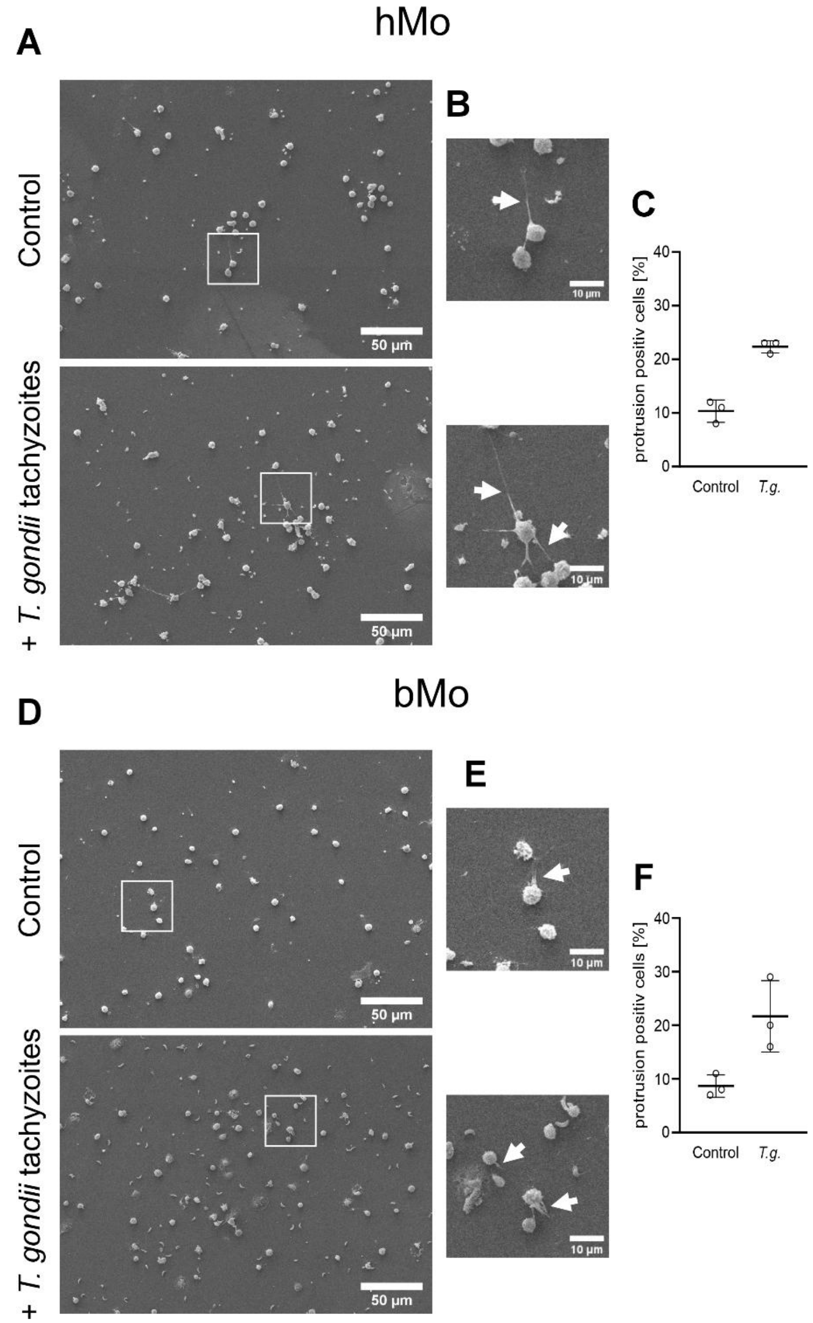

Supplementary Figure S4: Formation of cell protrusion (cytonemes, filopodia) in primary human and bovine monocytes exposed to *Toxoplasma gondii* tachyzoites.

Untreated (control) and parasite-exposed [4 h of co-incubation with *T. gondii tachyzoites* (MOI 1:4)] primary human (hMo) (**A**–**C**) and bovine monocytes (bMo) (**D**–**E**) were fixed after incubation and analyzed by scanning electron microscopy (SEM). Percentage of protrusion positive cells was determined from 5 – 10 overview images per condition (n = 3). Representative images of control and parasite-exposed cells with transparent boxes highlighting the counted structures (**A**,**D**), magnified crop examples of the transparent boxes with white arrows pointing to the protrusions (**B**,**E**), and mean percentage of protrusion positive cells and standard deviation (SD) (**C**,**F**) are shown. This Supplementary Figure S4 complements **Figure 1** in the main manuscript.

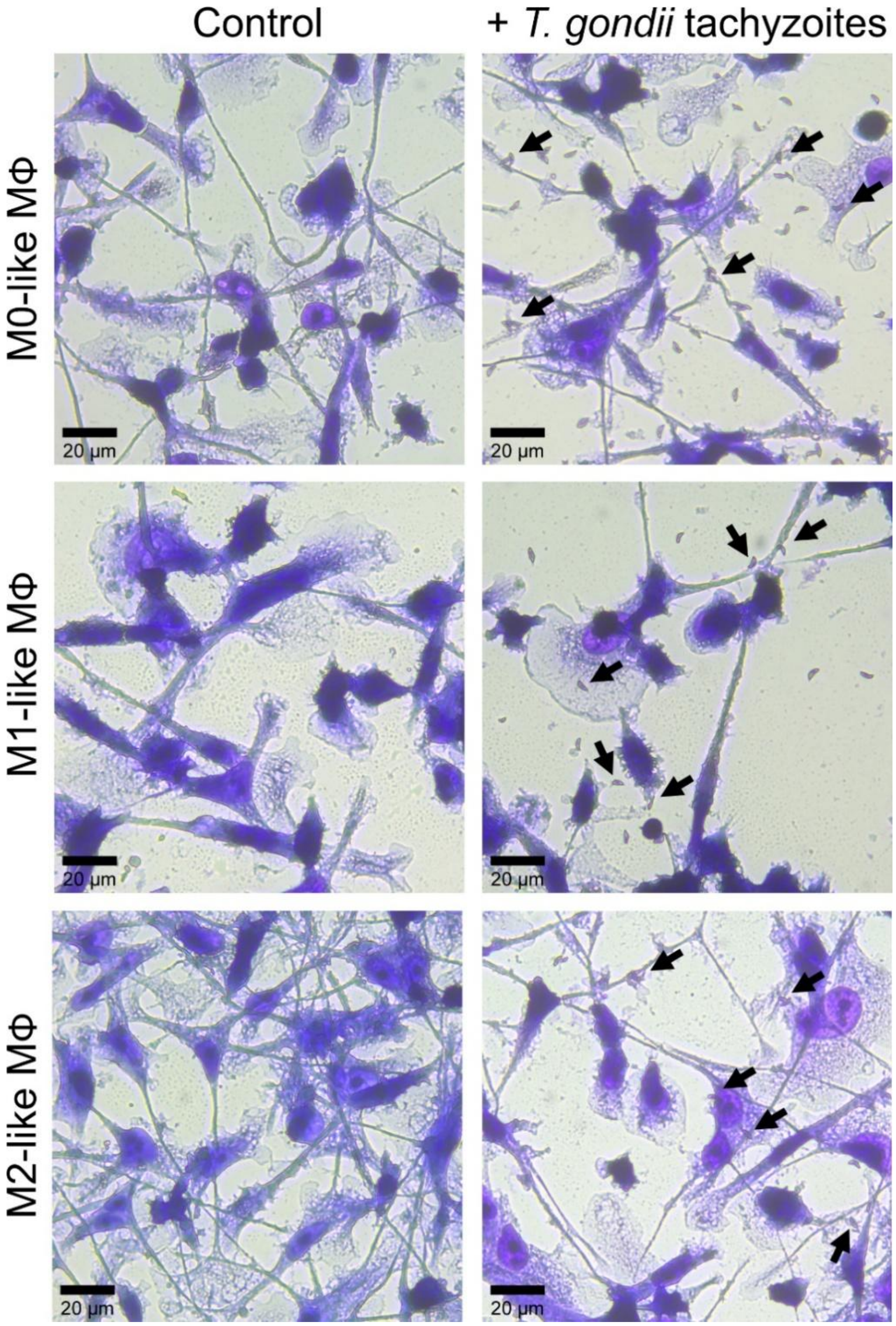

Supplementary Figure S5: Interaction of THP-1 cell-derived macrophages with *Toxoplasma gondii* tachyzoites.

Untreated (control) and parasite-exposed THP-1 cell-derived M0-, M1-, and M2-like macrophages (MΦ) were fixed after 4 h of co-incubation with *T. gondii* tachyzoites (+ *T. gondii* tachyzoites, MOI 1:4), stained by the Pappenheim method, and analysed by light microscopy. Interactions of THP-1 cell-derived macrophages with *T. gondii* tachyzoites are indicated by black arrows. Representative pictures from n = 3 independent experiments are shown.

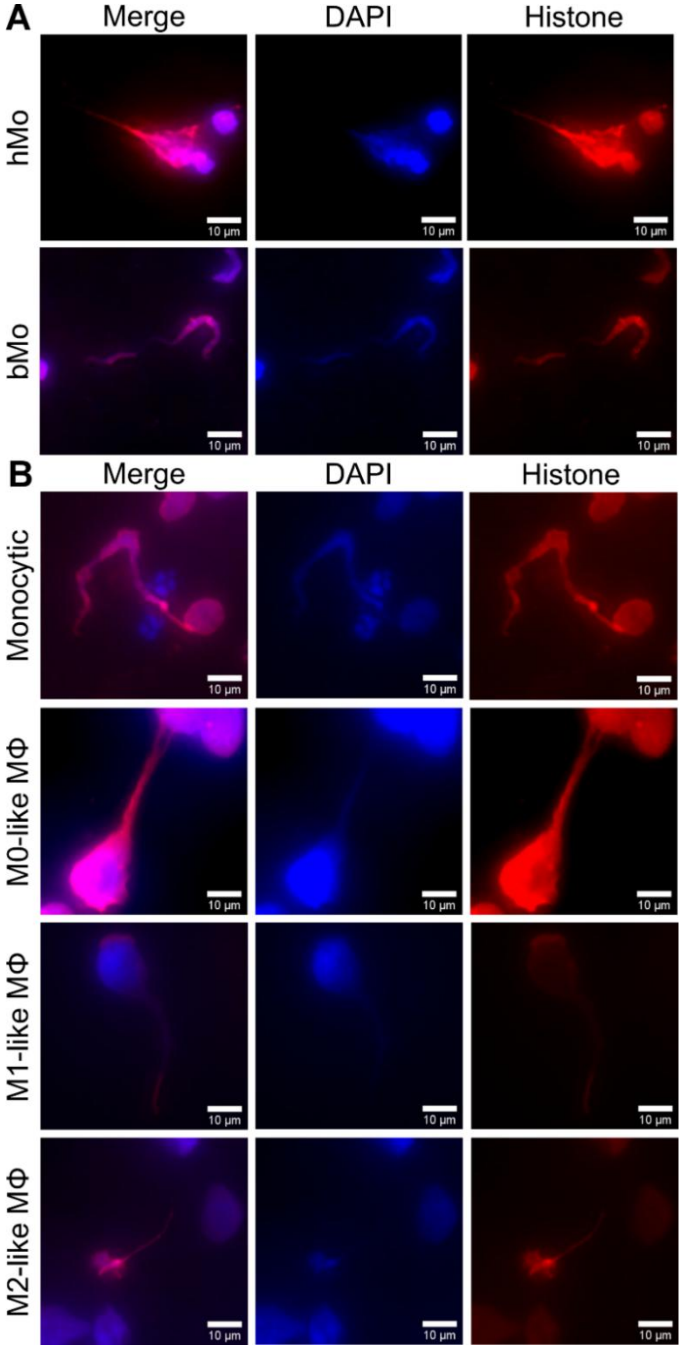

Supplementary Figure S6: Cropped and magnified images of the arrow-marked parasite-induced METs from Figure 3, showing individual and merged immunofluorescence channels.

Parasite-exposed primary human (hMo) and bovine monocytes (bMo) (**A**), monocytic THP-1 cells (monocytic) and THP-1 cell-derived M0-, M1-, and M2-like macrophages (MΦ) (**B**) were fixed after 4 h of co-incubation with *T. gondii tachyzoites* (MOI 1:4) and stained for DNA (DAPI, blue) and histone (red). Extracellular trap structure is shown in individual channels (DAPI, Histone); co-localization of DNA and histone in merged images (Merge) confirms monocyte/macrophage extracellular trap (MET) formation. This Supplementary Figure S6 complements **Figure 3** in the main manuscript.

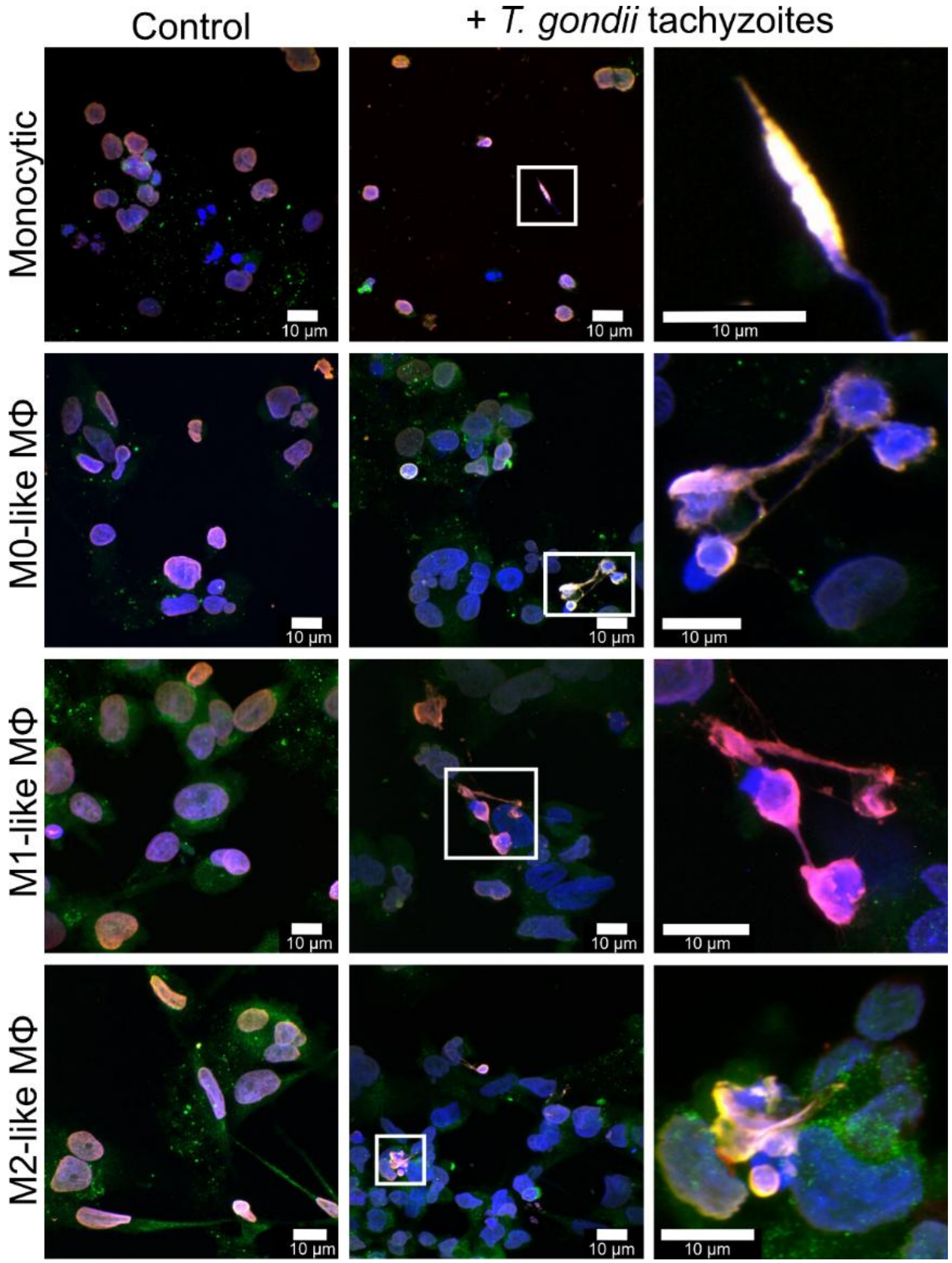

Supplementary Figure S7 (A): Extracellular trap marker detection in co-culture of *Toxoplasma gondii* tachyzoites with monocytic THP-1 cells and THP-1 cell-derived macrophages.

Untreated (control) and parasite-exposed monocytic THP-1 cells (monocytic) and THP-1 cell-derived M0-, M1-, and M2-like macrophages (MΦ) were fixed after 4 h of co-incubation with *T. gondii tachyzoites* (+ *T. gondii* tachyzoites, MOI 1:4) and stained for DNA (blue), histone (red), and myeloperoxidase (MPO, green). Extracellular traps, characterized by the co-localization of DNA, histone and MPO, are indicated by transparent boxes and magnified in the pictures shown on the right side. Representative pictures from n = 3 independent experiments are shown.

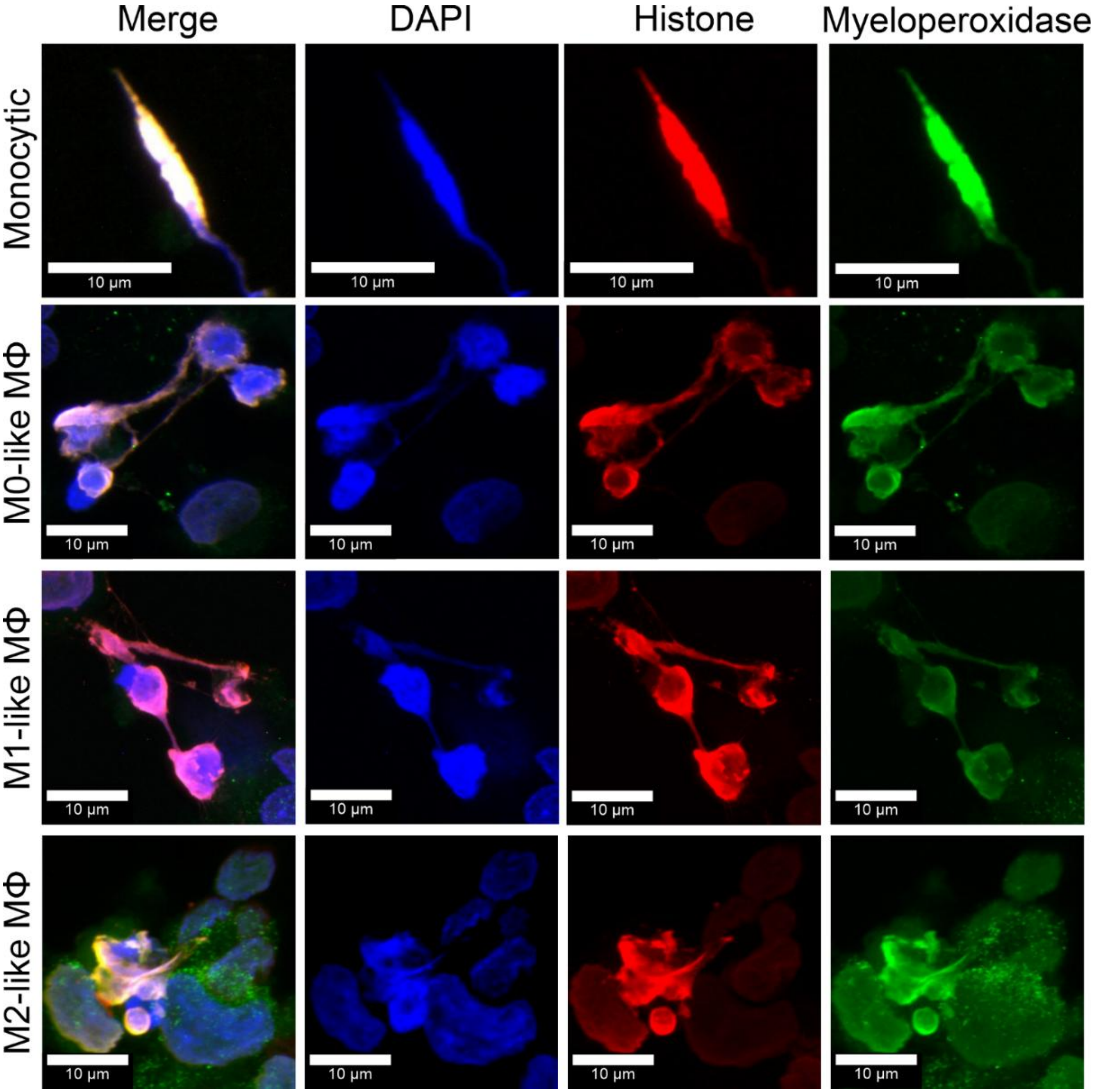

Supplementary Figure S7 (B): Cropped and magnified images of the transparent boxes marked parasite-induced extracellular traps from Supplementary Figure S7A, showing individual and merged immunofluorescence channels.

Parasite-exposed monocytic THP-1 cells (monocytic) and THP-1 cell-derived M0-, M1-, and M2-like macrophages (MΦ) were fixed after 4 h of co-incubation with *T. gondii tachyzoites* (MOI 1:4) and stained for DNA (DAPI, blue), histone (red), and myeloperoxidase (green). Extracellular trap structure is shown in individual channels (DAPI, Histone, myeloperoxidase); co-localization of DNA, histone and myeloperoxidase in merged images (Merge) confirms monocyte/macrophage extracellular trap (MET) formation.

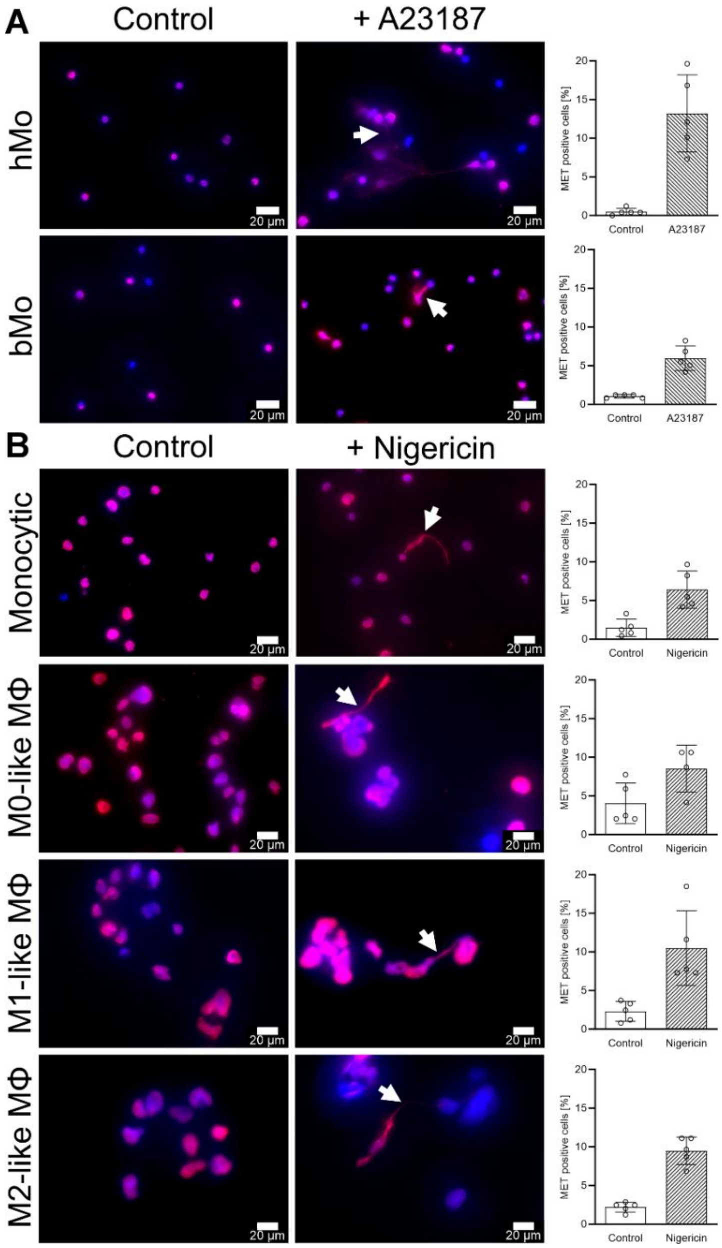

Supplementary Figure S8 (A): Extracellular trap marker detection in A23187- or nigericin-stimulated primary human and bovine monocytes, monocytic THP-1 cells and THP-1 cell-derived macrophages.

Primary human (hMo) and bovine monocytes (bMo) (**A**), monocytic THP-1 cells (monocytic) as well as THP-1 cell-derived M0-, M1-, and M2-like macrophages (MΦ) (**B**) were left untreated (control) or stimulated with A23187 [5 µM] (+ A23187) or nigericin [0.5µM] (+ nigericin). The cells were fixed after 4 h of incubation and stained for DNA (blue) and histone (red). Extracellular traps, which are characterized by the co-localization of DNA and histones, are indicated by white arrows. Percentage of monocyte/macrophage extracellular trap (MET) positive cells was determined ($n = 4 – 5$). The graphs show mean with standard deviation (SD). Statistical analysis was performed by comparing A23187/nigericin-treated cells with controls using the non-parametric Wilcoxon signed-rank test; no p-values below $p \leq 0.05$ were observed.

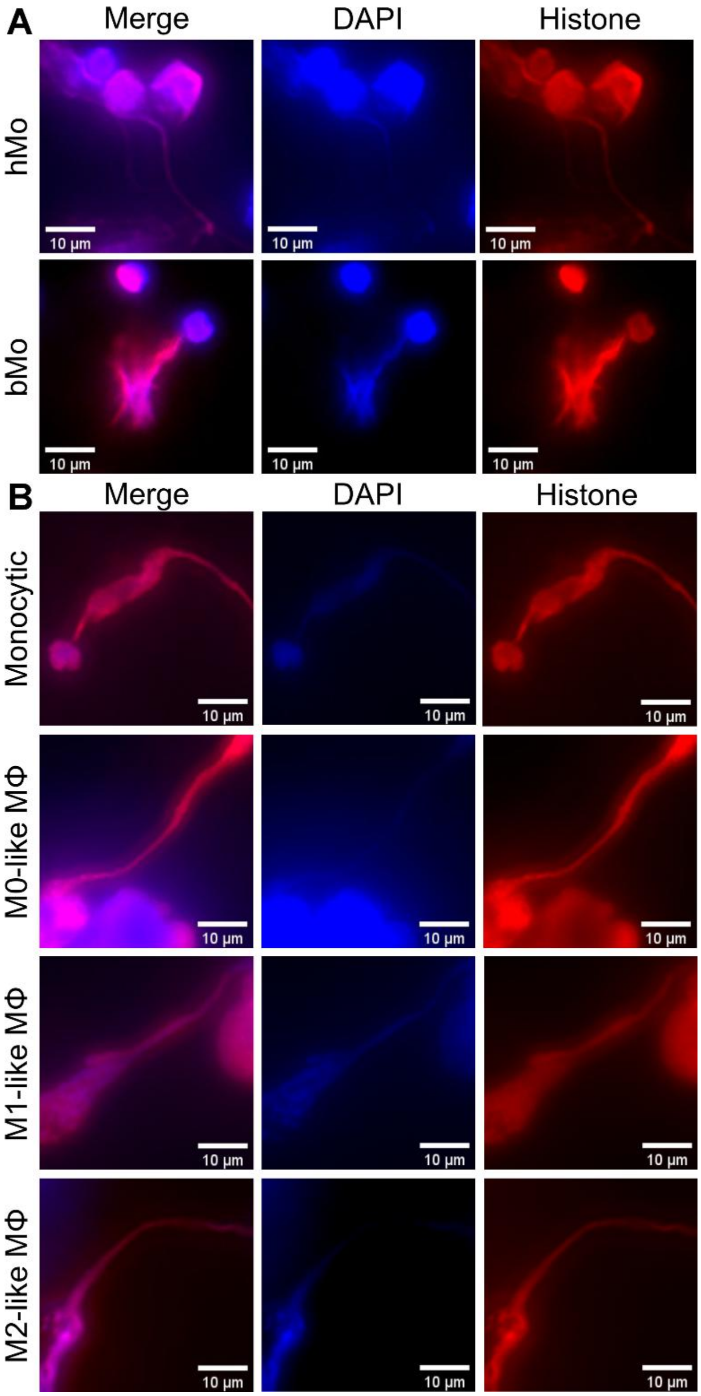

Supplementary Figure S8 (B): Cropped, magnified images from Supplementary Figure S8A displaying A23187- or nigericin-induced extracellular traps, with individual and merged immunofluorescence channels.

A23187 [5 µM]-treated primary human and bovine monocytes (**A**) and nigericin [0.5 µM]- treated monocytic THP-1 cells (monocytic) and THP-1 cell-derived M0-, M1-, and M2-like macrophages (MΦ) (**B**) were fixed after 4 h of incubation and stained for DNA (DAPI, blue) and histone (red). Extracellular trap structure is shown in individual channels (DAPI, Histone); co-localization of DNA and histone in merged images (Merge).

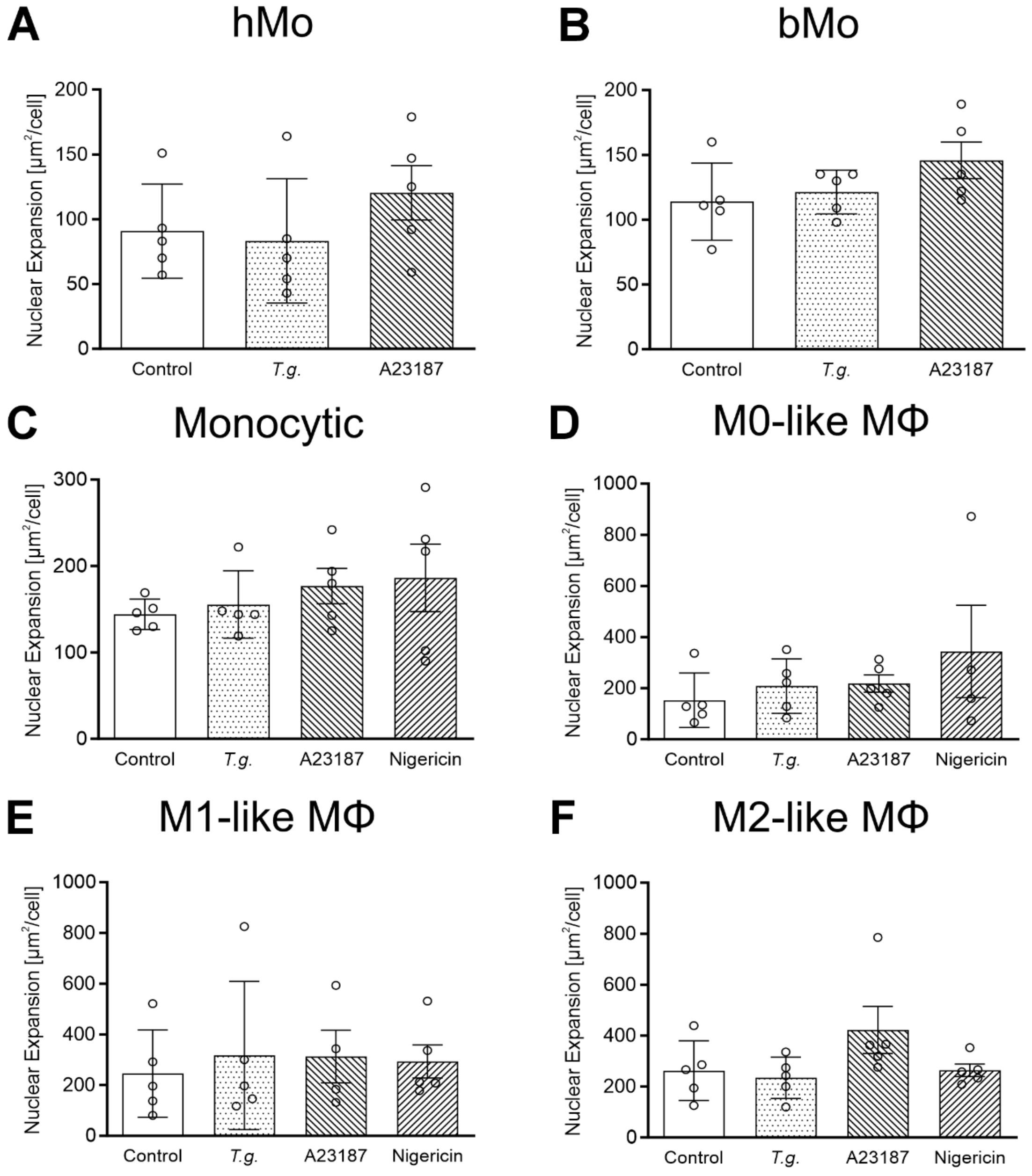

Supplementary Figure S9: Nuclear expansion of *Toxoplasma gondii*-exposed primary human and bovine monocytes, monocytic THP-1 cells and THP-1 cell-derived macrophages.

Primary human (hMo) (**A**) and bovine monocytes (bMo) (**B**), monocytic THP-1 cells (monocytic) (**C**) as well as THP-1 cell-derived M0- (**D**), M1- (**E**), and M2-like macrophages (MΦ) (**F**) were left untreated (control), exposed to *T. gondii* tachyzoites (*T.g.*, MOI 1:4) or treated with A23187 [5 µM] or nigericin [0.5 µM]. The cells were fixed after 4 h of incubation and stained for DNA. Nuclear expansion was calculated as the average of the DNA area per cell (n = 4 – 5). The graphs show the mean with standard deviation (SD). Statistical analysis was performed by comparing *T.g.*-exposed, A23187-treated or nigericin-treated cells, in each case compared to controls, using a two-way analysis of variance (ANOVA) with residual normality assessed to validate model assumptions, followed by the Bonferroni correction post-hoc pairwise comparison; no p-values $p \leq 0.05$ were observed.

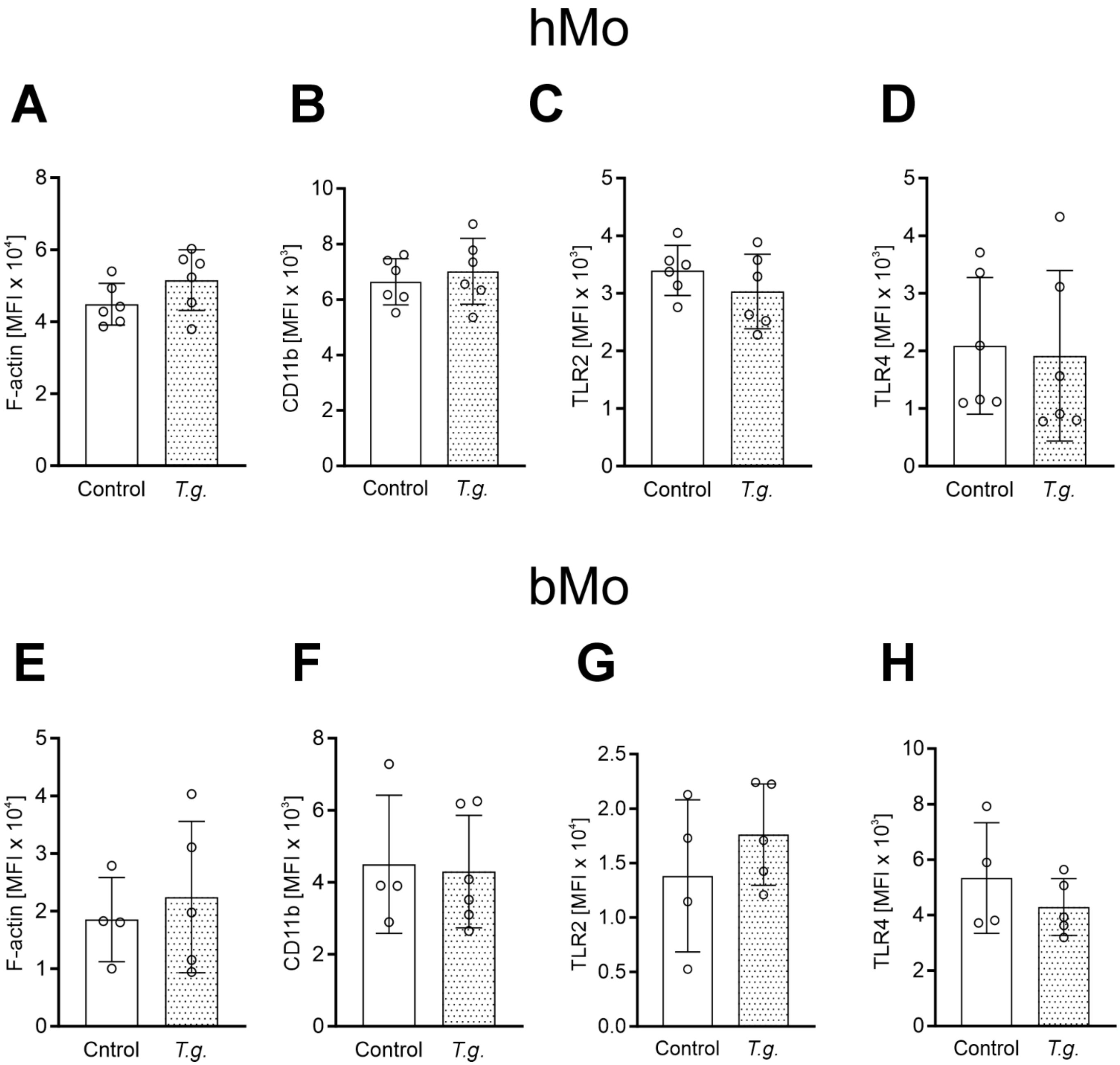

Supplementary Figure S10: Expression of F-actin, CD11b, Toll-like receptor (TLR) 2, and TLR4 by primary human and bovine monocytes.

Primary human (hMo) (**A** – **D**) and bovine monocytes (bMo) (**E** – **H**) were left untreated (control) or exposed to *Toxoplasma gondii* tachyzoites (*T.g.*, MOI 1:4) for 4 h each (n = 4 – 6). The expression of was analyzed using flow cytometry and is given as mean fluorescence intensity (MFI). The graphs show the mean with standard deviation (SD). Statistical analysis was performed by comparing *T.g.*-exposed cells with controls using the non-parametric Wilcoxon signed-rank test; no p-values below p ≤ 0.05 were observed.

Supplementary Table S1: Estimation of cell death of primary human monocytes, monocytic THP-1 cells and THP-1 cell-derived macrophages.

Figure	Cells, treatment	Cell death [%] mean ± SD	n
Figure 5A,B	hMo, -	2 ± 1	6
	hMo, *T.g.*	4 ± 2	6
	hMo, LPS	5 ± 6	6
	hMo, LPS, *T.g.*	3 ± 2	6
Figure 5C,D	Monocytic, -	2 ± 2	9
	Monocytic, *T.g.*	3 ± 2	9
	Monocytic, LPS	1 ± 1	9
	Monocytic, LPS, *T.g.*	2 ± 1	9
Figure 5E,F	M0-like MΦ, -	5 ± 3	6
	M0-like MΦ, *T.g.*	5 ± 4	6
	M0-like MΦ, LPS	4 ± 3	6
	M0-like MΦ, LPS, *T.g.*	5 ± 4	6
Figure 5G,H	M1-like MΦ, -	7 ± 6	6
	M1-like MΦ, *T.g.*	9 ± 7	6
	M1-like MΦ, LPS	5 ± 5	6
	M1-like MΦ, LPS, *T.g.*	5 ± 3	6
Figure 5I,J	M2-like MΦ, -	8 ± 5	6
	M2-like MΦ, *T.g.*	8 ± 5	6
	M2-like MΦ, LPS	6 ± 4	6
	M2-like MΦ, LPS, *T.g.*	5 ± 3	6

Primary human monocytes (hMo), monocytic THP-1 cells (monocytic) as well as THP-1 cell-derived M0-, M1-, and M2-like macrophages (MΦ) were left untreated (-), exposed to *T. gondii* tachyzoites (*T.g.*, MOI 1:4) or primed with lipopolysaccharide (LPS) [1 µg/ml] for 4 h each (n = 6 – 9). The cell death via lactate dehydrogenase (LDH) activity was quantified in cell culture supernatants. These data complement the experiments shown in **Figure 5**.

Supplementary Table S2: Measurements of the cytokines interleukin (IL)-1β and IL-6 as well as estimation of cell death in cell culture supernatants of primary human monocytes, monocytic THP-1 cells and THP-1 cell-derived macrophages.

Cells, treatment	IL-1β [pg/ml] mean ± SD	IL-6 [pg/ml] mean ± SD	Cell death [%] mean ± SD
hMo, -	2 ± 3, n = 6	0 ± 0, n = 6	2 ± 1, n = 5
hMo, A23187	34 ± 58, n = 6	0 ± 0, n = 6	26 ± 15, n = 5
hMo, LPS, BzATP	6994 ± 3682, n = 6	1209 ± 497, n = 6	1 ± 0, n = 5
hMo, LPS, nigericin#	7136 ± 4756, n = 6	450 ± 261, n = 6	13 ± 10, n = 2
Monocytic, -	1 ± 1, n = 9	8 ± 11, n = 9	2 ± 2, n = 9
Monocytic, A23187	4 ± 2, n = 9	9 ± 12, n = 9	40 ± 27, n = 9
Monocytic, nigericin*	2 ± 1, n = 9	5 ± 6, n = 9	29 ± 39, n = 6
Monocytic, LPS, BzATP	55 ± 56, n = 9	11 ± 11, n = 9	2 ± 3, n = 9
Monocytic, LPS, nigericin#	74 ± 94, n = 9	11 ± 11, n = 9	78 ± 23, n = 3
M0-like MΦ, -	3 ± 2, n = 6	5 ± 4, n = 6	5 ± 3, n = 6
M0-like MΦ, A23187	7 ± 2, n = 6	5 ± 5, n = 6	67 ± 24, n = 6
M0-like MΦ, nigericin*	4 ± 1, n = 6	4 ± 3, n = 6	18 ± 13, n = 3
M0-like, LPS MΦ, BzATP	91 ± 46, n = 6	329 ± 67, n = 6	3 ± 3, n = 6
M0-like MΦ, LPS, nigericin#	452 ± 414, n = 6	138 ± 121, n = 6	40 ± 29, n = 3
M1-like MΦ, -	104 ± 34, n = 6	8 ± 6, n = 6	7 ± 6, n = 6
M1-like MΦ, A23187	671 ± 110, n = 6	11 ± 8, n = 6	80 ± 26, n = 6
M1-like MΦ, nigericin*	270 ± 111, n = 6	8 ± 6, n = 6	27 ± 24, n = 3
M1-like MΦ, LPS, BzATP	1171 ± 414, n = 6	649 ± 128, n = 6	9 ± 8, n = 6
M1-like MΦ, LPS, nigericin#	1746 ± 978, n = 6	366 ± 318, n = 6	34 ± 24, n = 3
M2-like MΦ, -	3 ± 2., n = 6	7 ± 6, n = 6	8 ± 5, n = 6
M2-like MΦ, A23187	9 ± 3, n = 6	7 ± 6, n = 6	60 ± 36, n = 6
M2-like MΦ, nigericin*	5 ± 2, n = 6	7 ± 4, n = 6	16 ± 11, n = 3
M2-like MΦ, LPS, BzATP	64 ± 51, n = 6	150 ± 58, n = 6	6 ± 3, n = 6
M2-like MΦ, LPS, nigericin#	142 ± 111, n = 6	78 ± 58, n = 6	17 ± 15, n = 3

Primary human monocytes (hMo), monocytic THP-1 cells (monocytic) as well as THP-1 cell-derived M0-, M1-, and M2-like macrophages (MΦ) were left untreated (-), treated with A23187 [5 µM] or nigericin [0.5 µM] (*) for 4 h, or primed with lipopolysaccharide (LPS) [1 µg/ml] for 3.5 h followed by treatment with 2'/3'-O-(4-benzoylbenzoyl)adenosine-5'-triphosphate, tri(triethylammonium) salt (BzATP) [200 µM] or nigericin [25 µM] (#) for 40 min each (n = 6 – 9). The concentrations of IL-1β and IL-6 were measured in cell culture supernatants via ELISA. Cell death was estimated via measurement of the release of lactate dehydrogenase (LDH) into the cell culture medium. These data complement the experiments shown in **Figure 5**.
